# Supplementary material for: Notch Signaling Mediates Secondary Senescence
Source: Cell Rep. 2019 Apr 23;27(4):997–1007.e5. doi: 10.1016/j.celrep.2019.03.104 (PMC6486482; doi:10.1016/j.celrep.2019.03.104)
Supplement: Document S1. Figures S1–S4 [file mmc1.pdf]

Cell Reports, Volume *27*

Supplemental Information

Notch Signaling Mediates Secondary Senescence

Yee Voan Teo, Nattaphong Rattanavirotkul, Nelly Olova, Angela Salzano, Andrea Quintanilla, Nuria Tarrats, Christos Kiourtis, Miryam Müller, Anthony R. Green, Peter D. Adams, Juan-Carlos Acosta, Thomas G. Bird, Kristina Kirschner, Nicola Neretti, and Tamir Chandra

Figure S1

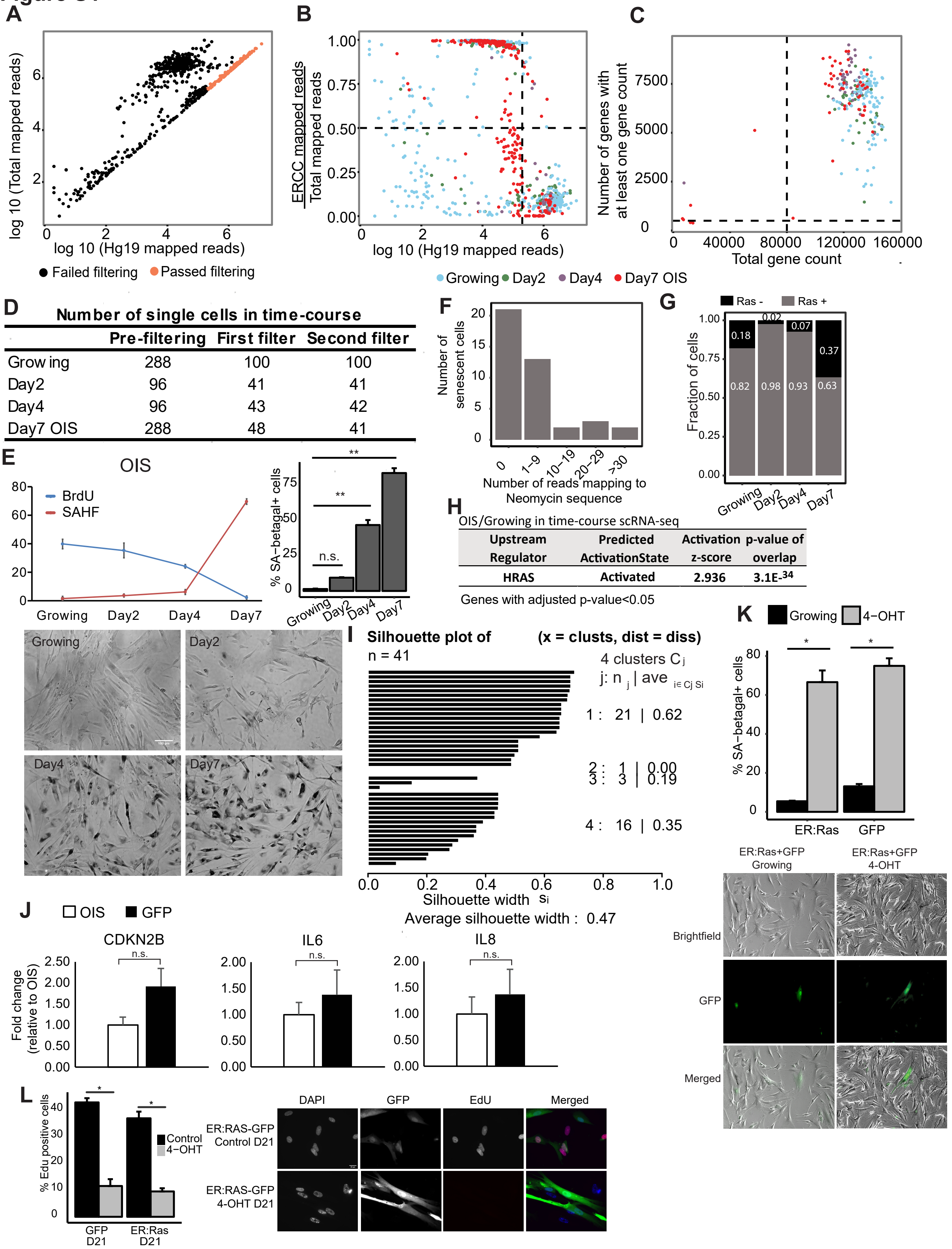

D

Number of single cells in time-course

	Pre-filtering	First filter	Second filter
Growing	288	100	100
Day2	96	41	41
Day4	96	43	42
Day7 OIS	288	48	41

H

OIS/Growing in time-course scRNA-seq

Upstream Regulator	Predicted ActivationState	Activation z-score	p-value of overlap
HRAS	Activated	2.936	3.1E-34

Genes with adjusted p-value<0.05

Figure S1, Related to Figure 1.

(A,B) Filtering according to total mapped reads. Cells with less than 200,000 human aligned reads and with a ratio of ERCC RNA spike-in control aligned reads to total aligned reads that is greater than 0.5 were removed.

(C) The second filtering step was performed to retain cells that have greater than 80,000 total gene counts and at least 500 genes with at least one count. Cell were normalised by downsampling to 200,000 aligned reads for downstream analysis.

(D) The number of cells that passed the filtering step in (A) and (B)

(E) BrdU, SAHF and SA-Beta galactosidase counts in ER:Ras fibroblasts. Days indicated time of tamoxifen treatment. Error bars are SEM, n=3 for each time point. $F[3,8] = 234.8$, $p<0.001$; **$p < 0.001$ using one-way ANOVA with Tukey's test. Scale bar 100µm.

(F) Number of reads aligning to the neomycin sequence from the pLNCX2-ER-ras_neo construct in senescent single cells in the time-course experiment.

(G) Fraction of cells that are RasV12+ in each condition in the time-course experiment.

(H) IPA analysis of OIS/growing in time-course scRNA-seq.

(I) Silhouette plot to assess the quality of clustering. The average silhouette width was 0.47.

(J) Box plots for gene expression of *CDKN2B* (n=3), *IL6* (n=3), and *IL8* (n=3) mRNA measured by qPCR in OIS and GFP cells. Unpaired Student's t-test showed no significant difference in senescent markers expression between OIS and GFP cells. Error bars represent SEM.

(K) SA-Beta galactosidase counts in OIS and GFP cell s. (OIS t = 10.199, df = 2.0096, p= 0.009; GFP t = 15.239, df = 2.3673, p= 0.002 using unpaired Student's t-test). Scale bar 100µm.

(L) Bar plots showing EdU incorporation in GFP cells co-cultured with ER:Ras cells after 21 days as proportion of all cells scored. Error bars are displayed as SEM; **p<0.001, *p<0.05. Representative images are shown. Scale bar 20µm. (ER:Ras t = -9.899, df = 2.86 68, p= 0.0024; GFP t = 10.395, df = 3.3348, p= 0.0012 using unpaired Student's t-test) (n=3 per experiment).

Figure S2

A Top activated and shared upstream regulator assessed by IPA

Time-course
(Secondary senescence / OIS)

Upstream Regulator	Activation z-score	p-value of overlap
decitabine	2.058	1.16E-13
TGFB1	1.839	1.64E-12
Brd4	0.788	2.55E-13
TP53	0.653	1.44E-14
ERBB2	0.611	2.01E-13
forskolin	0.488	4.7E-13
D-glucose	0.271	1.45E-12
ERK	-0.193	1E-15
KRAS	-0.805	1E-13
EGF	-0.835	2.57E-16
lipopolysaccharide	-0.97	1.01E-13
HRAS	-3.116	1.09E-17

Co-culture
(Secondary senescence / OIS)

Upstream Regulator	Activation z-score	p-value of overlap
PD98059	4.571	5.34E-29
U0126	3.069	5.91E-29
dexamethasone	2.646	2.65E-33
TGFB1	2.622	1.3E-48
MYCN	-2.054	1.07E-22
Cg	-2.253	2.03E-24
EGF	-2.322	4.52E-22
EGFR	-2.617	3.39E-26
KRAS	-2.989	3.37E-24
PDGF BB	-3.101	4.29E-36
TNF	-3.435	2.34E-29
HRAS	-4.235	8.86E-37

Genes with adjusted p-value<0.05

B

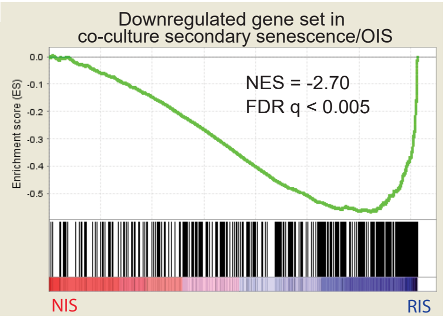

Leading edge genes: STC1, MMP1, SERPINB2, CDCP1, NRG1, SLC16A6, TFPI1, TMEM158, CSF3, IL8, MT2A, HMGA1, IL1B

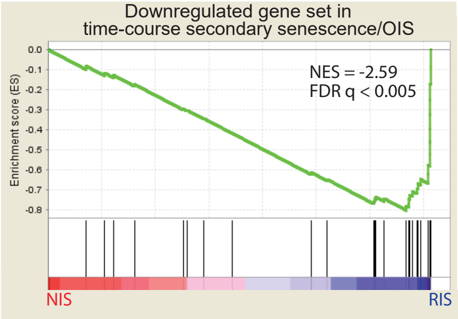

Leading edge genes: MMP3, STC1, MMP1, SERPINB2, SLC16A6, TFP12, IL2B,

C

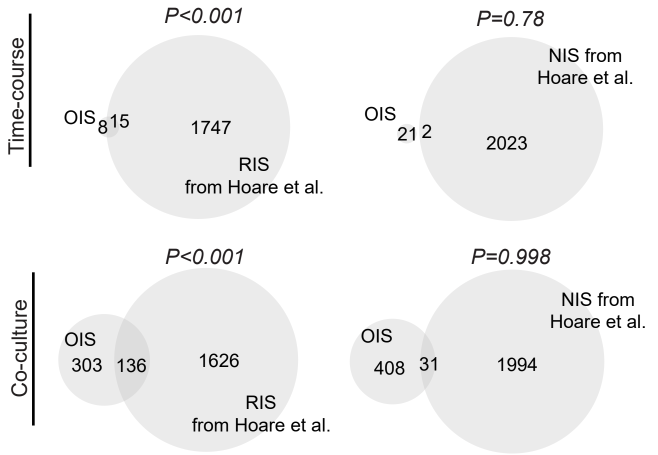

Figure S2, Related to Figure 2

(A) IPA analysis of the two senescence clusters from time-course and co-culture scRNA-seq. Red indicates activated upstream regulator and blue indicates inhibited upstream regulator.

(B) GSEA was used to assess the enrichment of secondary and primary senescence (OIS) DE genes in Hoare et al.'s NIS and RIS log2FC preranked genes. NES and FDR are shown.

(C) Venn diagrams overlapping expression signatures from top panel: time-course and bottom panel: co-culture experiments with NIS signature genes (OIS: OIS/Secondary senescence upregulated genes; NIS: Hoare et al.'s NIS/RIS upregulated genes; RIS: Hoare et al.'s RIS/NIS upregulated genes)

Figure S3

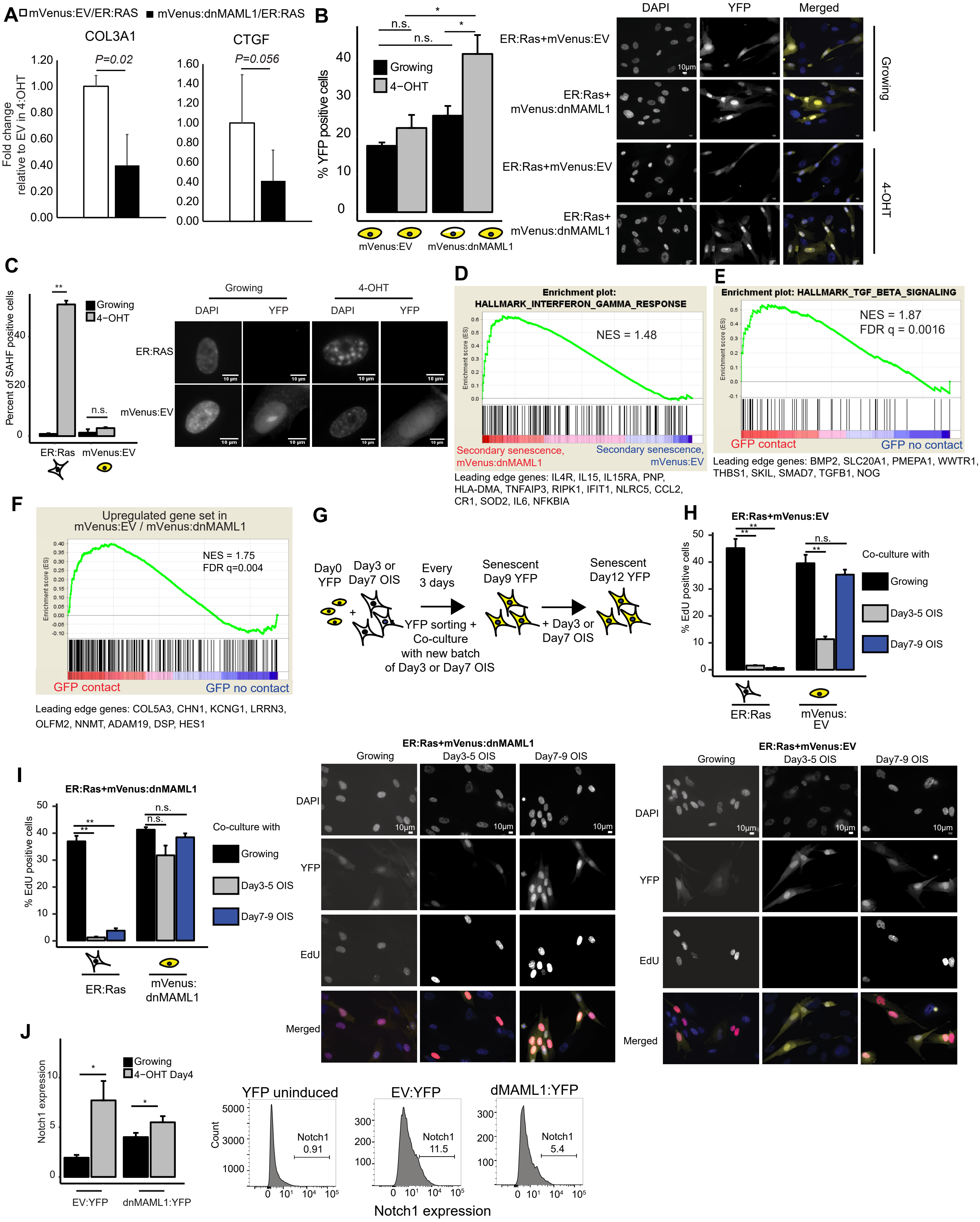
A
mVenus:EV/ER:RAS
mVenus:dnMAML1/ER:RAS
COL3A1
P=0.02
CTGF
P=0.056
Fold change relative to EV in 4:OHT
B
Growing
4−OHT
% YFP positive cells
n.s.
*
mVenus:EV
mVenus:dnMAML1
DAPI
YFP
Merged
ER:Ras+mVenus:EV
ER:Ras+ mVenus:dnMAML1
C
Percent of SAHF positive cells
ER:Ras
mVenus:EV
ER:RAS
D
Enrichment plot: HALLMARK_INTERFERON_GAMMA_RESPONSE
NES = 1.48
Secondary senescence, mVenus:dnMAML1
Secondary senescence, mVenus:EV
Leading edge genes: IL4R, IL15, IL15RA, PNP, HLA-DMA, TNFAIP3, RIPK1, IFIT1, NLRC5, CCL2, CR1, SOD2, IL6, NFKBIA
E
Enrichment plot: HALLMARK_TGF_BETA_SIGNALING
NES = 1.87
FDR q = 0.0016
GFP contact
GFP no contact
Leading edge genes: BMP2, SLC20A1, PMEPA1, WWTR1, THBS1, SKIL, SMAD7, TGFB1, NOG
F
Upregulated gene set in mVenus:EV / mVenus:dnMAML1
NES = 1.75
FDR q=0.004
GFP contact
GFP no contact
Leading edge genes: COL5A3, CHN1, KCNG1, LRRN3, OLFM2, NNMT, ADAM19, DSP, HES1
G
Day0 YFP
Day3 or Day7 OIS
Every 3 days
YFP sorting + Co-culture with new batch of Day3 or Day7 OIS
Senescent Day9 YFP
+ Day3 or Day7 OIS
Senescent Day12 YFP
H
ER:Ras+mVenus:EV
% EdU positive cells
Co-culture with
Growing
Day3-5 OIS
Day7-9 OIS
ER:Ras
mVenus: EV
I
ER:Ras+mVenus:dnMAML1
ER:Ras+mVenus:EV
Growing
Day3-5 OIS
Day7-9 OIS
DAPI
YFP
EdU
Merged
ER:Ras
mVenus: dnMAML1
J
Growing
4−OHT Day4
Notch1 expression
EV:YFP
dnMAML1:YFP
YFP uninduced
Notch1 0.91
EV:YFP
Notch1 11.5
dMAML1:YFP
Notch1 5.4
Count
Notch1 expression

Figure S3, Related to Figure 3

(A) Bar plot showing the expression of *CTGF* (n=6) and *COL3A1* (n=3) genes in EV or dnMAML1 cells compared to ER:Ras senescent cells by qPCR. (COL3A1: t=5.3405, df=2.4861, $p=0.02$; CTGF: t=2.2104, df=8.4894, $p=0.056$ using unpaired Student's t- test). Error bars represent SEM.

(B) Bar plot denoting the proportion of growing (black) or senescent (grey) mVenus cells with dnMAML1 or EV as proportion of all cells scored. Error bars are displayed as SEM; $F[3,8] = 10.05$, $p<0.05$ using one-way ANOVA with Tukey's test (n=3 for each condition). Representative images of mVenus cells and cells stained with DAPI are shown on the right. Scale bar 10μm.

(C) SAHF counts in OIS and secondary senescent (unpaired Student's t-test, ER:Ras t=-34.05, df=2.12, ** $p<0.01$; mVenus:EV t=-1.23, df=2.28, $p=0.32$; n=3 for each condition). Representative images are shown on the right.

(D) GSEA pre-ranked test for enrichment of interferon gamma response in mVenus:dnMAML1 identified as secondary senescence by scmap.

(E) GSEA pre-ranked test for enrichment of TGF-beta signalling in GFP contact compared to GFP no contact cells. mVenus:dnMAML1 identified as secondary senescence by scmap.

(F) GSEA pre-ranked test for enrichment of mVenus:EV signature genes in GFP contact/GFP no contact upregulated gene set.

(G) Schematic representation of co- culturing mVenus cells with Day 3 or Day 7 OIS cells.

(H) Bar plot showing EdU incorporation in OIS or mVenus:EV cells in growing (black), co- culture with Day 3 OIS (grey) or Day 7 OIS cells (blue). Error bars are displayed as SEM; $F[5,18] = 144.4$, $p<0.001$ using one-way ANOVA with Tukey's test (n=3 for each except for Day 3 OIS (n=6)). Representative images are shown below the bar plot. Scale bar 10μm.

(I) Bar plot showing EdU incorporation in OIS or mVenus:dnMAML1 cells in growing (black), co-culture with Day 3 OIS (grey) or Day 7 OIS cells (blue). Error bars are displayed as SEM. $F[5,24] = 58$, $p<0.001$ using one-way ANOVA with Tukey's test (n=3 for all conditions except for Day3 OIS (n=6)). **$p<0.001$, *$p<0.05$. Representative images are shown on the right of the bar plot. Scale bar 10μm. **j.** Barplot showing the upregulation of NOTCH1 on the cell surface of mVenus:EV and mVenus:dnMAML1 cells 4 days after co-culture with ER:Ras compared to non-co-cultured, growing mVenus:EV (mVenus:EV t = -3.27, df = 2.01, p-value = 0.041; mVenus:dnMAML1 t = -3.29, df =3.03, p-value = 0.023 using one-sided t- test). Error bars represent SEM. Representative FACs plots showing NOTCH1 staining of YFP uninduced fibroblasts and YFP:EV and YFP:dnMaml1 at 4 days of co-culture.

Figure S4

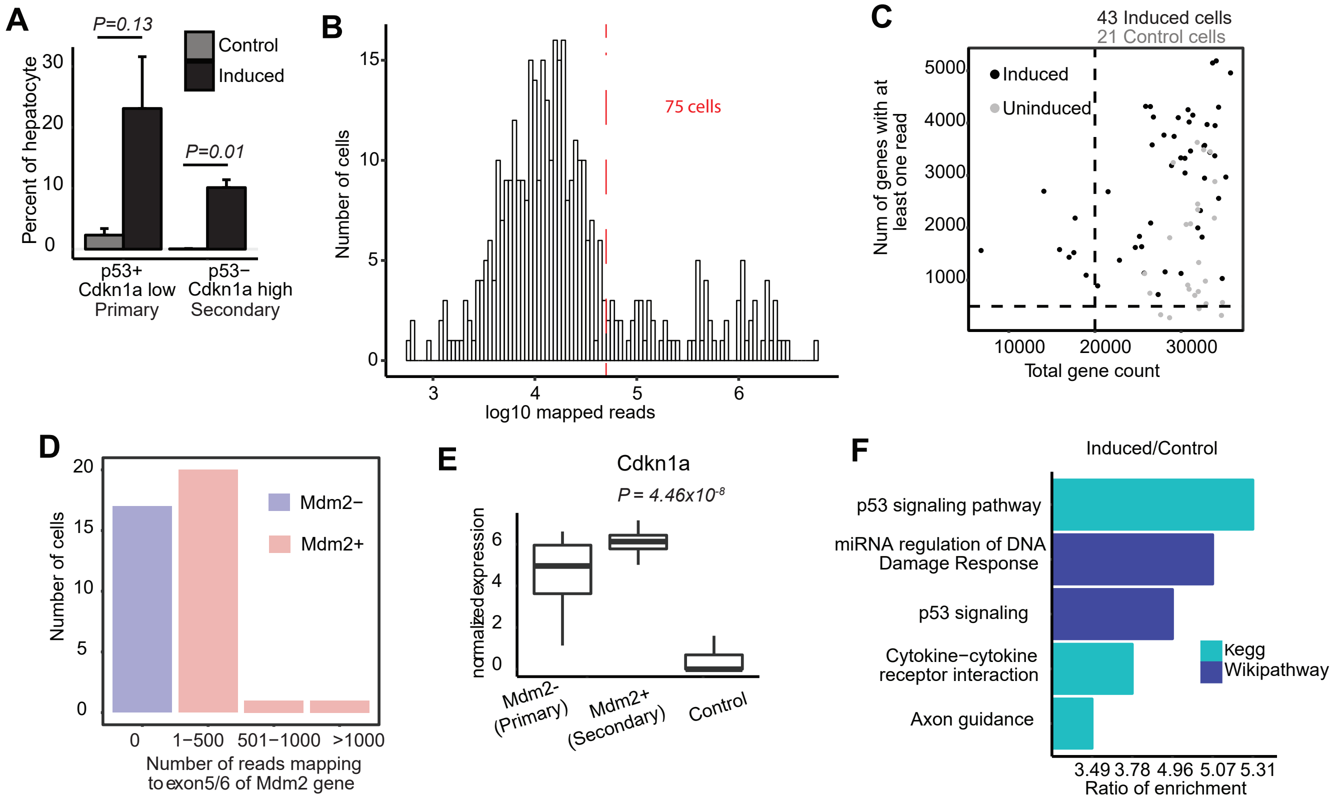
A
P=0.13
P=0.01
Control
Induced
Percent of hepatocyte
30
20
10
0
p53+ Cdkn1a low Primary
p53− Cdkn1a high Secondary
B
75 cells
Number of cells
15
10
5
0
3
4
5
6
log10 mapped reads
C
43 Induced cells
21 Control cells
Induced
Uninduced
Num of genes with at least one read
5000
4000
3000
2000
1000
10000
20000
30000
Total gene count
D
Mdm2−
Mdm2+
Number of cells
20
15
10
5
0
0
1−500
501−1000
>1000
Number of reads mapping to exon5/6 of Mdm2 gene
E
Cdkn1a
P = 4.46x10-8
normalized expression
6
4
2
0
Mdm2- (Primary)
Mdm2+ (Secondary)
Control
F
Induced/Control
p53 signaling pathway
miRNA regulation of DNA Damage Response
p53 signaling
Cytokine−cytokine receptor interaction
Axon guidance
Kegg
Wikipathway
3.49
3.78
4.96
5.07
5.31
Ratio of enrichment

Figure S4, Related to Figure 4

(A) Bar graph denoting the percentage of primary and secondary hepatocytes (Primary: t=2.4241, df=2.0641, p-value = 0.1324; Secondary: t=7.7563, df=2.0053, p=0.0161 using unpaired Student's t-test).

(B) Histogram with the number of induced and control cells is plotted against log mapped reads. 75 single cells with at least 50,000 aligned reads are downsampled to 50,000 reads.

(C) Dot plot with the number of genes with at least one read to total gene count for induced (black) and control (grey) cells. Cells with a total gene count of more than 20,000 and 500 genes detected were retained.

(D) 17 *Mdm2*- cells were identified as cells with no reads mapping to exon5/6 of *Mdm2* gene and 22 *Mdm2*+ cells contained reads mapping to the exons.

(E) Box plots showing the expression of *Cdkn1a* in induced cells relative to control ($p=4.46x10^{-18}$). The top and bottom bounds of the boxplot correspond to the 75 and 25th percentile, respectively. p-values were obtained using differential analysis in SCDE.

(F) Pathway analysis for induced/uninduced hepatocytes. Kegg pathways are shown in turquoise and Wikipathways in blue.
