# Supplementary material for: Notch Signaling Mediates Secondary Senescence
Source: Cell Rep. 2019 Apr 23;27(4):997–1007.e5. doi: 10.1016/j.celrep.2019.03.104 (PMC6486482; doi:10.1016/j.celrep.2019.03.104)
Supplement: Document S2. Article plus Supplemental Information [file mmc6.pdf]

Cell Reports

Report

Notch Signaling Mediates Secondary Senescence

Graphical Abstract

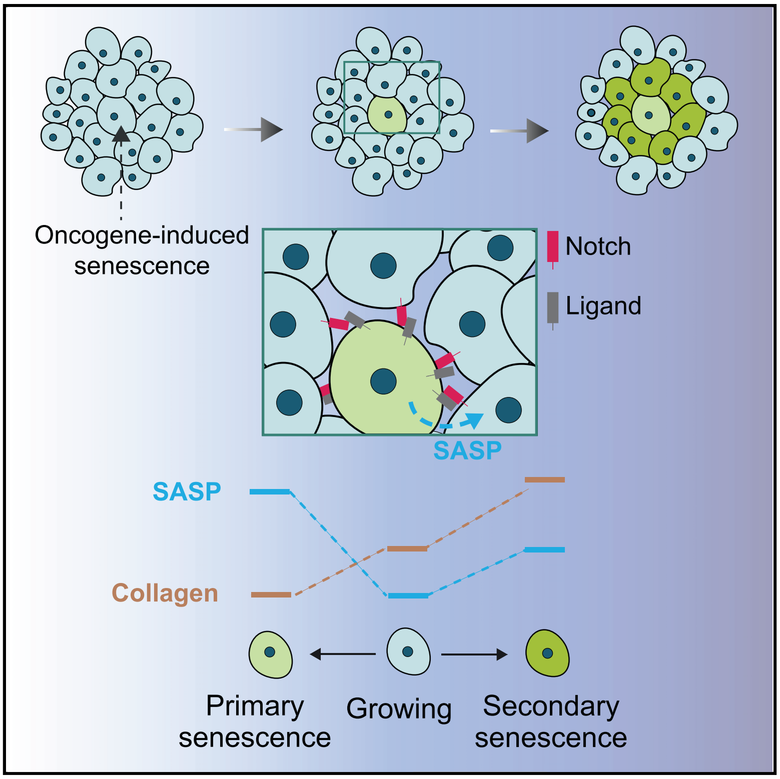

Highlights

- Primary senescence and secondary senescence are distinct molecular endpoints
- Secondary Ras-induced senescence has a composite SASP, Notch-induced signature
- Notch signaling is an essential driver of secondary senescence
- Notch blunts the senescence-associated secretory phenotype in secondary senescence

Authors

Yee Voan Teo, Nattaphong Rattanavirotkul, Nelly Olova, ..., Kristina Kirschner, Nicola Neretti, Tamir Chandra

Correspondence

kristina.kirschner@glasgow.ac.uk (K.K.), nicola_neretti@brown.edu (N.N.), tamir.chandra@igmm.ed.ac.uk (T.C.)

In Brief

Teo et al. found that secondary senescence *in vitro* and *in vivo* requires Notch, rather than SASP alone, as previously thought. The authors show that primary and secondary senescence are distinct molecular endpoints. A blunted secretory phenotype and the induction of fibrillar collagens in secondary senescence point toward functional diversification.

Teo et al., 2019, Cell Reports *27*, 997–1007
April 23, 2019 © 2019 The Authors.
https://doi.org/10.1016/j.celrep.2019.03.104

Cell Reports
Report

OPEN ACCESS
CellPress

Notch Signaling Mediates Secondary Senescence

Yee Voan Teo,[2,10] Nattaphong Rattanavirotkul,[1,9,10] Nelly Olova,[1] Angela Salzano,[1] Andrea Quintanilla,[1] Nuria Tarrats,[1] Christos Kiourtis,[3,4] Miryam Müller,[4] Anthony R. Green,[6] Peter D. Adams,[3,4,7] Juan-Carlos Acosta,[1] Thomas G. Bird,[4,5] Kristina Kirschner,[3,*] Nicola Neretti,[2,8,*] and Tamir Chandra[1,11,*]
[1]MRC Human Genetics Unit, MRC Institute of Genetics and Molecular Medicine, The University of Edinburgh, Edinburgh, UK
[2]Department of Molecular Biology, Cell Biology and Biochemistry, Brown University, Providence, RI 02903, USA
[3]Institute of Cancer Sciences, University of Glasgow, Glasgow G61 1BD, UK
[4]CRUK Beatson Institute, Glasgow G61 1BD, UK
[5]MRC Centre for Inflammation Research, University of Edinburgh, Edinburgh EH164TJ, UK
[6]Wellcome/MRC Cambridge Stem Cell Institute and Department of Haematology, Jeffrey Cheah Biomedical Centre, University of Cambridge, Cambridge CB2 0AW, UK
[7]Sanford Burnham Prebys Medical Discovery Institute, La Jolla, CA 92037, USA
[8]Center for Computational Molecular Biology, Brown University, Providence, RI 02906, USA
[9]Wellcome Sanger Institute, Hinxton, Cambridgeshire CB10 1SA, UK
[10]These authors contributed equally
[11]Lead Contact
*Correspondence: kristina.kirschner@glasgow.ac.uk (K.K.), nicola_neretti@brown.edu (N.N.), tamir.chandra@igmm.ed.ac.uk (T.C.)
https://doi.org/10.1016/j.celrep.2019.03.104

SUMMARY

Oncogene-induced senescence (OIS) is a tumor suppressive response to oncogene activation that can be transmitted to neighboring cells through secreted factors of the senescence-associated secretory phenotype (SASP). Currently, primary and secondary senescent cells are not considered functionally distinct endpoints. Using single-cell analysis, we observed two distinct transcriptional endpoints, a primary endpoint marked by Ras and a secondary endpoint marked by Notch activation. We find that secondary oncogene-induced senescence *in vitro* and *in vivo* requires Notch, rather than SASP alone, as previously thought. Moreover, Notch signaling weakens, but does not abolish, SASP in secondary senescence. Global transcriptomic differences, a blunted SASP response, and the induction of fibrillar collagens in secondary senescence point toward a functional diversification between secondary and primary senescence.

INTRODUCTION

Cellular senescence is a stress response, resulting in stable cell cycle arrest, tumor suppression, aging, and wound healing (Adams, 2009; Campisi, 2013; Jun and Lau, 2010; van Deursen, 2014). Aberrant activation of the Ras oncogene triggers oncogene-induced senescence (OIS), conferring a precancerous state (Di Micco et al., 2006; Serrano et al., 1997). OIS is an *in vivo* tumor suppressor mechanism (Braig et al., 2005; Xue et al., 2007) with the p53 and Rb/p16 pathways as major mediators of senescence induction and maintenance (Kirschner et al., 2015; Serrano et al., 1997). OIS is characterized by multiple phenotypical changes, such as heterochromatic foci (Adams, 2007; Chandra and Kirschner, 2016; Criscione et al., 2016; Kirschner et al., 2015; Narita et al., 2003) and the senescence-associated secretory phenotype (SASP) (Acosta et al., 2008; Coppé et al., 2008; Kuilman et al., 2008). Through the secretion of extracellular matrix proteases, interleukins, and chemokines, OIS cells recruit immune cells, mediating their own clearance. SASP has been implicated in cancer initiation (Watanabe et al., 2017) by creating an inflammatory pro-tumorigenic microenvironment. SASP factors play a role in cellular reprogramming (Mosteiro et al., 2016; Ritschka et al., 2017) and contribute to aging and tissue degeneration (Osorio et al., 2012; Soria-Valles et al., 2019). SASP acts in a paracrine fashion to induce secondary senescence in surrounding cells (Acosta et al., 2013). Paracrine secondary senescence is thought to enhance immune surveillance and to act as a failsafe mechanism minimizing chances of retaining damaged cells (Acosta et al., 2013; Kuilman et al., 2008; Nelson et al., 2012). Recently, ectopic Notch pathway activation has been implicated as an intermediate phenomenon during primary senescence induction, resulting in a distinct secretome (Hoare et al., 2016). The role of Notch in secondary OIS mediation remains undescribed.

Here, we use single-cell RNA sequencing (scRNA-seq) to decipher the heterogeneity within OIS populations. Our single-cell experiments reveal two distinct transcriptional endpoints in primary senescence, separated by their activation of Notch, with secondary senescent cells uniformly progressing to an endpoint characterized by Notch activation *in vivo* and *in vitro*. We confirm Notch-mediated senescence as an essential mediator of secondary, juxtacrine senescence in OIS.

Primary and Secondary Senescence Have Distinct Transcriptomes

To investigate dynamic changes and cell-cell heterogeneity in OIS, we performed a scRNA-seq time course experiment

This is an open access article under the CC BY license (http://creativecommons.org/licenses/by/4.0/).

before and after 2, 4, and 7 days of RasV12 induction, using H-RasG12V-induced IMR90 (ER:IMR90) fibroblasts (Young et al., 2009) and the Smart-Seq2 protocol (Picelli et al., 2014) (Figure 1A). After stringent filtering (Figure 1B; Figures S1A–S1D; Table S1), we obtained a final cell count of 100/288 for day 0, 41/96 for day 2, 42/96 for day 4, and 41/288 for day 7 for downstream analysis (Figure S1D). To confirm a senescence phenotype at day 7, we profiled bromodeoxyuridine (BrdU) incorporation (37/390 cells [9%]), senescence associated heterochromatic focis (SAHF; 265/390 cells [68%]), and senescence-associated beta-galactosidase (SA-Beta Gal) (428/523 cells [82%]) (Figure S1E). To assess time-dependent changes in the transcriptome, we ordered cells along a pseudo-temporal trajectory based on differential gene expression between growing and senescence (adjusted $p < 0.05$; Table S2 (Figure 1C) (Kharchenko et al., 2014). Using Monocle2 (Qiu et al., 2017), we found a continuous progression from growing to senescence, with days 2 and 4 cells as intermediates and two distinct senescent populations (Figure 1C), suggesting two facultative, alternative endpoints. To determine whether RasV12 activation led to the split into two senescence populations (Figure 1C), we overlaid RasV12 expression onto the monocle plot (Figures 1B and 1C; Figures S1F and S1G; Table S3). RasV12-expressing cells (Figure 1C; Ras+, round symbols) progressed to both senescence endpoints with a 21:4 skew toward the cluster designated OIS. Fibroblasts without detectable RasV12 expression uniformly progressed to the cluster tentatively designated secondary senescence, suggesting it as the obligate endpoint (cross symbols, Ras−; Figure 1C; Fisher's exact test, 1.64×10^{-6}). Our inability to detect RasV12 in a subset of senescent cells suggests that senescence was induced as a secondary event. We verified HRAS as one of the top predicted upstream regulators for the senescence top population ($p = 3.1 \times 10^{-34}$) by using Ingenuity pathway analysis (IPA, QIAGEN; Figure S1H). We confirmed a senescence phenotype for both populations by upregulation of key senescence genes (Figure 1D) cyclin-dependent kinase 1a (*CDKN1A*) and cyclin-dependent kinase inhibitor 2b (*CDKN2B*) and SASP factors interleukin 8 (*IL8*), interleukin 6 (*IL6*), and interleukin 1B (*IL1B*) ($p < 0.05$ for all genes; Figure 1D). To verify two major senescence populations transcriptome-wide, we used a consensus clustering approach, SC3 (Kiselev et al., 2017), with the number of clusters determined by silhouette plot (Rousseeuw, 1987) (Figure S1I). SC3 detected two senescence clusters largely overlapping with the subpopulations obtained by Monocle2 (cluster 1 16/21 or 76% RasV12+ cells, cluster 4 11/15 or 73% RasV12− cells), supporting the notion that the split into two senescence populations is based on the absence or presence of RasV12 (Figure 1E). To verify that populations observed are due to primary OIS and secondary senescence, we co-cultured ER:IMR90 with IMR90:GFP fibroblasts (10:1), where secondary senescence is induced in IMR90:GFP-positive cells (Acosta et al., 2013). We generated scRNA-seq data before and 7 days after RasV12 activation by using the 10× Genomics Chromium (Figure 1F).

Senescence was confirmed on sorted populations by qPCR (Figure S1J) and SA-Beta Gal staining for primary and secondary senescent cells (Figure S1K). Cells were annotated based on GFP, RasV12 expression, and the G > T mutation of *Ras* gene (Figure 1G). We identified three distinct clusters using Seurat and Sparcl (Butler et al., 2018; Witten and Tibshirani, 2010), namely growing (blue dots), secondary senescence (GFP positive, black dots) and OIS (RasV12 positive, red dots), with significant enrichment for the OIS and secondary senescence populations (chi-square test, $p = 4.1 \times 10^{-14}$; Figure 1H). The secondary senescence cluster also contained a minor population of RasV12-expressing cells. This mirrors our earlier findings, confirming two facultative senescence endpoints for primary RasV12 senescent cells, with GFP-positive secondary senescent cells showing a uniform distribution. Senescence genes were upregulated in both senescent clusters, including *CDKN1A*, *CDKN2B*, and *IL8* (Figure 1I; Table S2) and long-term stable cell cycle arrest confirmed at 21 days post co-culture (Figure S1L). When overlaying transcriptomes of the time course and the co-culture experiments, a significant number of cells identified as OIS and secondary senescence (GFP and part of RasV12) clustered together (Figure 1J; chi-square test, $p < 0.05$). The co-clustering by senescent signatures was achieved despite the data being generated by 10× or Smart-Seq2. In summary, we identified two major transcriptional endpoints in primary OIS, whereas secondary senescent cells were uniformly assigned.

Paracrine senescence is thought to be the main effector mechanism for secondary, cell extrinsic senescence induction (Acosta et al., 2013; Kuilman et al., 2008). To test if the secondary senescence cluster is explained by a paracrine signature, we overlaid bulk RNA-seq data (Acosta et al., 2013). Although we found a significant overlap with paracrine genes (hypergeometric test: paracrine/OIS and time course secondary senescence/OIS (Ras−/Ras+) $p < 0.001$; paracrine/OIS and 10× secondary senescence/OIS $p < 0.001$, 10× secondary senescence/OIS and time course secondary senescence/OIS (Ras−/Ras+) $p < 0.001$; Figure 1K; Table S4), a large fraction of genes shared between our two single cell experiments remained unexplained, suggesting the involvement of additional pathways in secondary senescence.

The Transcriptome of Secondary and a Subset of Primary Senescent Cells Is Characterized by Notch

Because the secondary senescence clusters were only partially characterized by a paracrine senescence signature, we explored consistent differences between the secondary senescence and the primary OIS clusters. We assessed the most differentially expressed genes and detected fibrillar collagens (collagen 1A1, 3A1, and 5A2; Figure 2A). Downregulation of fibrillar collagens is consistently observed in senescence (Hoare et al., 2016), but they failed to downregulate in the secondary senescence cluster (Table S2; Figure 2A). A similar failure to downregulate collagens was reported in a specialized primary senescence phenotype, induced by ectopic, temporal activation of Notch (Hoare et al., 2016). The same report suggested that the secretome in RasV12-induced senescence was regulated by CCAAT-enhancer-binding protein beta (CEBPB), with Notch-induced senescence relying on transforming growth factor beta (TGFB) (Figure 2B) (Hoare et al., 2016). Several lines of evidence identify a notch-induced senescence (NIS) signature in the secondary senescence cluster. First, IPA pathway analysis

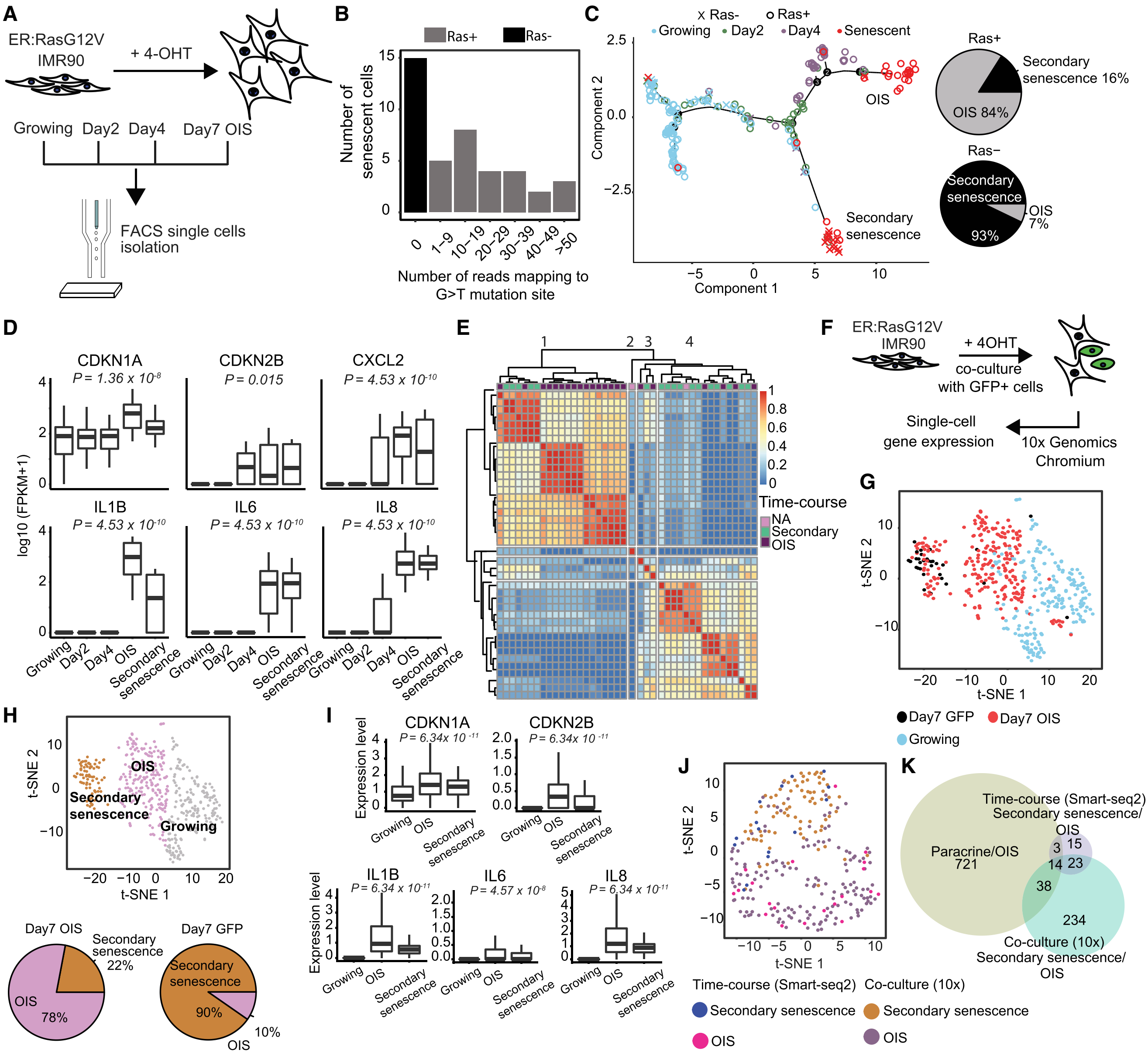

Figure 1. Secondary Senescent Cells Only Partially Resemble Paracrine-Induced Senescence

(A) Schematic representation of the time course experiment.

(B) Number of senescent cells with reads mapping to the G > T mutation site of *RAS* gene.

(C) Monocle2 plot for time course experiment. The presence of the mutated *RAS* gene is indicated. Pie charts for the percentage of Ras+/Ras− cells in the top and bottom clusters.

(D) Boxplots for the expression of senescence genes in the time course experiment. The top and bottom bounds of the boxplot correspond to the 75th and 25th percentile, respectively. p values were obtained using differential analysis in SCDE.

(E) Unsupervised clustering using SC3 for senescent cells. Cells were annotated as either OIS (top senescence branch, purple), secondary senescence (bottom branch, green), or NA (neither, pink).

(F) Schematic representation of the co-culture experiment.

(G) t-Distributed Stochastic Neighbor Embedding (tSNE) visualization of co-culture scRNA-seq.

(H) tSNE visualization of single cells grouped into 3 clusters.

(I) Boxplots for the expression of senescence genes in the co-culture experiment. The top and bottom bounds of the boxplot correspond to the 75th and 25th percentile, respectively. p values were obtained using differential analysis in SCDE.

(J) Integration analysis of the two senescence clusters from time course and co-culture experiments.

(K) Overlap of differentially expressed (DE) genes between paracrine/OIS, time course, and co-culture experiments.

Related to Figure S1 and Tables S1–S4.

OPEN ACCESS
CellPress

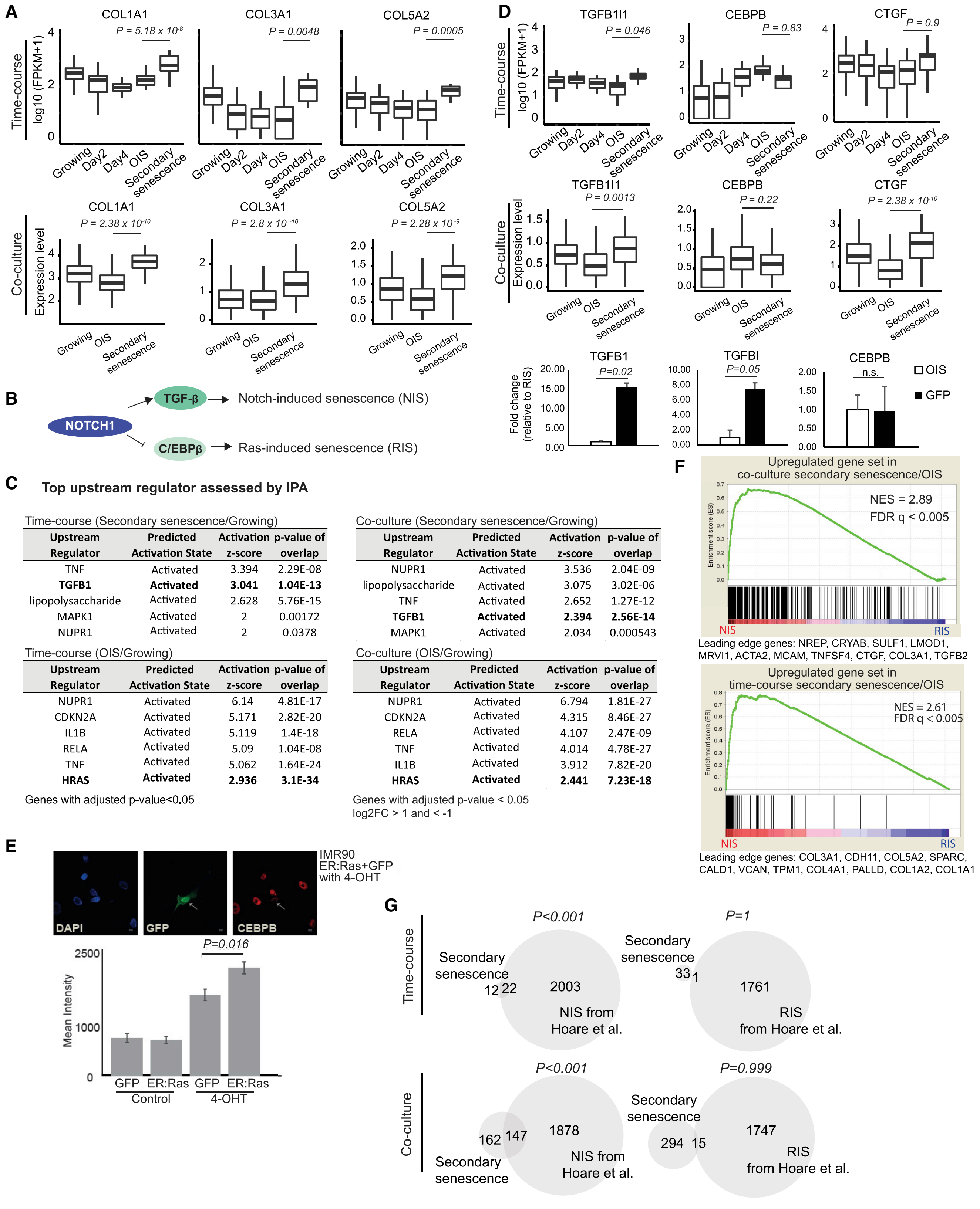

C Top upstream regulator assessed by IPA

Time-course (Secondary senescence/Growing)

Upstream Regulator	Predicted Activation State	Activation z-score	p-value of overlap
TNF	Activated	3.394	2.29E-08
**TGFB1**	**Activated**	**3.041**	**1.04E-13**
lipopolysaccharide	Activated	2.628	5.76E-15
MAPK1	Activated	2	0.00172
NUPR1	Activated	2	0.0378

Time-course (OIS/Growing)

Upstream Regulator	Predicted Activation State	Activation z-score	p-value of overlap
NUPR1	Activated	6.14	4.81E-17
CDKN2A	Activated	5.171	2.82E-20
IL1B	Activated	5.119	1.4E-18
RELA	Activated	5.09	1.04E-08
TNF	Activated	5.062	1.64E-24
**HRAS**	**Activated**	**2.936**	**3.1E-34**

Genes with adjusted p-value<0.05

Co-culture (Secondary senescence/Growing)

Upstream Regulator	Predicted Activation State	Activation z-score	p-value of overlap
NUPR1	Activated	3.536	2.04E-09
lipopolysaccharide	Activated	3.075	3.02E-06
TNF	Activated	2.652	1.27E-12
**TGFB1**	**Activated**	**2.394**	**2.56E-14**
MAPK1	Activated	2.034	0.000543

Co-culture (OIS/Growing)

Upstream Regulator	Predicted Activation State	Activation z-score	p-value of overlap
NUPR1	Activated	6.794	1.81E-27
CDKN2A	Activated	4.315	8.46E-27
RELA	Activated	4.107	2.47E-09
TNF	Activated	4.014	4.78E-27
IL1B	Activated	3.912	7.82E-20
**HRAS**	**Activated**	**2.441**	**7.23E-18**

Genes with adjusted p-value < 0.05
log2FC > 1 and < -1

(legend on next page)

identifies TGFB1 as exclusively activated in the secondary senescence clusters compared to growing or the primary OIS (Figure 2C). In contrast, RELA and IL1B pathways, regulators of the CEBPB transcriptome, were differentially activated in primary OIS clusters (Figure 2C). Consistent with our RasV12 annotation, HRAS was exclusively activated in primary OIS (Figure 2C; Figure S2A). Second, we profiled candidate genes involved in Notch signaling and TGFB activation. When plotting TGFB-induced transcript 1, (*TGFB1I1*) with Notch-target connective tissue growth factor (*CTGF*) and *CEBPB*, we identified a significant ($p < 0.05$) upregulation of *CTGF* and *TGFB1I1* genes in the secondary senescence cluster with a simultaneous downregulation of CEBPB, significant on the protein but not mRNA level (Figures 2D and 2E; p = 0.016), resembling the TGFB and CEBPB bias in NIS. This bias was confirmed by qPCR (Figure 2D; TGFB1 p = 0.02, TGFBI p = 0.05).

Third, we applied an unbiased genome-wide analysis. We calculated the enrichment of NIS and Ras-induced senescence (RIS) signatures in the primary OIS and secondary senescence transcriptomes by using gene set enrichment analysis (GSEA) (Subramanian et al., 2005) on ranked transcriptome differences between NIS and RIS (Figure 2F). Secondary senescence signatures from the time course and co-culture experiments were highly enriched in NIS (normalized enrichment score [NES] = 2.61, false discovery rate [FDR] < 0.005 for time course; NES = 2.89, FDR < 0.005 for co-culture experiments; Figure 2F). Primary OIS transcriptomes showed an enrichment for RIS (Figure S2B). Finally, we interrogated the extent of NIS in secondary senescence by comparing the most differentially regulated genes (adjusted $p < 0.05$) between RIS and NIS. We found a significant enrichment of NIS genes in our secondary senescence transcriptome in the time course and co-culture experiments, with primary OIS signature being enriched for RIS (Figures 2F and 2G; Figures S2B and S2C). In summary, our data identify a pronounced NIS signature in secondary senescence and in a subset of primary senescent cells as an alternative endpoint to OIS.

NIS Is a Secondary Senescence Effector Mechanism during OIS

We next established Notch signaling as an effector mechanism in secondary senescence. We generated IMR90 fibroblasts with compromised Notch signaling by introducing a dominant-negative form of mastermind-like protein 1 fused to mVenus (mVenus:dnMAML1) or empty vector (mVenus:EV) control and co-cultured with ER:Ras IMR90 cells in the presence of tamoxifen (Figure 3A). At day 7 co-culture, mVenus:dnMAML1 compared to mVenus:EV cells exhibited lower expression of extracellular matrix gene *COL3A1* (p = 0.02) and Notch target *CTGF* (p = 0.056; Figure S3A) as measured by qPCR, confirming impaired Notch signaling. Several lines of evidence show causal involvement of Notch signaling in secondary senescence. First, we scored mVenus (YFP) signal between mVenus:dnMAML1 and mVenus:EV cells at day 0 (growing) and day 7 co-culture with ER:Ras. At day 7, we observed significantly more mVenus:dnMAML1 compared to mVenus:EV cells (p = 0.01), suggesting that primary OIS cells have less secondary senescence effect on neighboring cells when harboring perturbed Notch signaling (Figure S3B). No significant difference in mVenus-positive cells was observed in growing mVenus:EV compared to mVenus:dnMAML1 cells (p = 0.38), showing that the dnMAML1 itself does not affect cell numbers (Figure S3B). Second, we scored EdU incorporation between mVenus:dnMAML1 and mVenus:EV cells at days 0 and 7 (Figure 3B). At day 7, we observed significantly more EdU incorporation in mVenus:dnMAML1 compared to mVenus:EV cells (p = 0.01), with day 7 mVenus:dnMAML1 cells showing similarly high levels of EdU incorporation as growing mVenus:dnMAML1 and growing mVenus negative ER:Ras conditions (p = 0.997 and p = 0.08), suggesting that the induction of secondary senescence was abolished due to Notch perturbation (Figure 3B). As expected, ER:Ras cells showed low levels of EdU incorporation at day 7 tamoxifen (p = 0.01 for ER:Ras/mVenus:dnMAML1 co-culture and p = 0.0005 for ER:Ras/mVenus:EV co-culture; Figure 3B).

Third, we investigated SAHF in primary OIS and secondary senescence. Primary OIS cells displayed SAHF as expected ($p = 4.437 \times 10^{-6}$; Figure S3C). Secondary senescent cells (mVenus:EV) did not show significant SAHF formation when compared to OIS (p = 0.32; Figure S3C). This is consistent with published data where impaired Notch signaling partially suppresses SAHF formation in primary senescence (Parry et al., 2018). In summary, we show that Notch signaling mediates secondary senescence *in vitro*.

Figure 2. Secondary Senescence Comprises NIS Signature in the Majority of Cells

(A) Boxplots for the expression of genes *COL1A1*, *COL3A1*, and *COL5A2* in the time course and co-culture experiments ($p < 0.05$). The top and bottom bounds of the boxplots correspond to the 75th and 25th percentile, respectively. p values were obtained using differential analysis in SCDE.

(B) Model suggesting NIS and RIS are regulated by Notch1 through TGFB and CEBPB, respectively.

(C) IPA analysis of the two senescence clusters from the time course and co-culture experiments relative to growing.

(D) Boxplots for the expression of *TGFB1I1*, *CTGF*, and *CEBPB* genes in the time course (top) and co-culture experiments (middle). The top and bottom bounds of the boxplot correspond to the 75th and 25th percentile, respectively. p values were obtained using differential analysis in SCDE. Bar graphs denoting expression of *TGFB1* (n = 6), *TGFBL* (n = 6), and *CEBPB* (n = 3) mRNA as measured by qPCR in OIS and GFP cells (bottom) (TGFB1: t = −3.2317, df = 5.5117, p = 0.02; TGFBI: t = −2.2567, df = 9.8141, p = 0.05; CEBPB: t = 0.068192, df = 3.2294, p = 0.95, unpaired Student's t test. Error bars represent SEM).

(E) Representative image of GFP (secondary senescence) and CEBPB (red) immunofluorescence in the co-culture experiment. Mean intensity for primary (ER:Ras) and secondary senescent cells (GFP) was measured (p = 0.016, unpaired Student's t test). Error bars are displayed as SEM.

(F) GSEA plots for the enrichment of secondary and primary OIS DE genes (time course and co-culture experiments) in Hoare et al. (2016) NIS and RIS log2FC preranked genes. Normalized enrichment score (NES) and false discovery rate (FDR) are shown.

(G) Venn diagrams overlapping expression signatures from time course (top) and co-culture (bottom) with NIS signature genes. (Secondary senescence: Secondary senescence/OIS upregulated genes; NIS: Hoare et al. (2016) NIS/RIS upregulated genes; RIS: Hoare et al. (2016) RIS/NIS upregulated genes.)

Related to Figure S2 and Table S4.

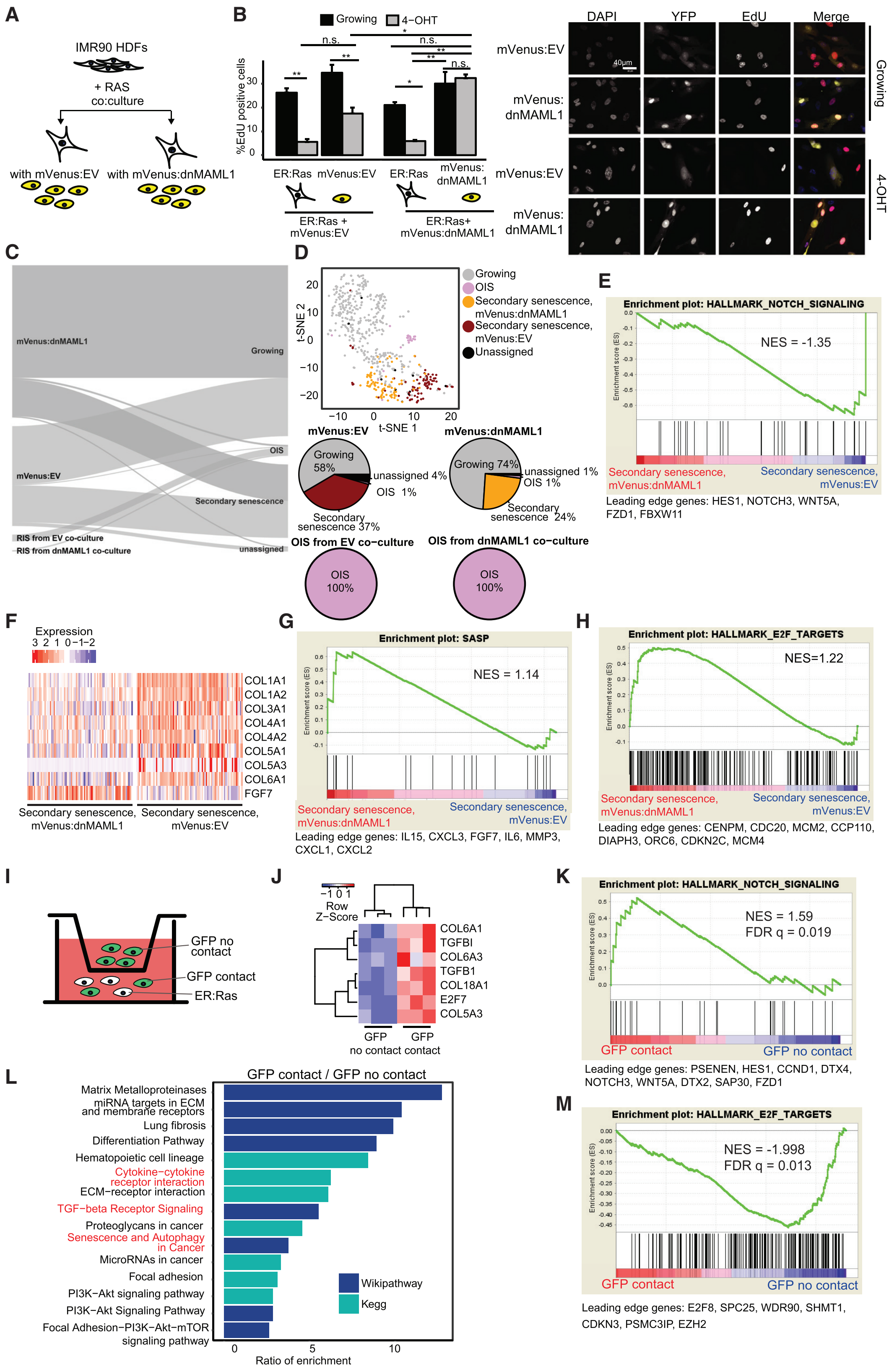

(legend on next page)

To establish transcriptional differences between secondary senescence with and without Notch signaling, we generated scRNA-seq data from IMR90 mVenus:EV and mVenus:dnMAML1 co-cultures with ER:Ras IMR90 at day 7 tamoxifen. To integrate this dataset with our previous secondary senescence transcriptomes (Figure 1H), we projected the mVenus:EV and mVenus:dnMAML1 using Scmap (Kiselev et al., 2018). Scmap clearly matches all primary senescent cells containing RasV12 to the OIS population (Figure 3C) and identifies significantly more secondary senescence cells in mVenus:EV compared to mVenus:dnMaml1 (Figure 3C; 37% versus 24%, chi-square test, $p = 0.00062$), confirming a role of Notch in secondary senescence. To explore transcriptomic differences between secondary senescence, we plotted all cells using Seurat, which separated mVenus:EV and mVenus:dnMAML1 into distinct secondary senescence clusters (Figure 3D). We confirmed differences in the activation of Notch pathway between mVenus:EV and mVenus:dnMaml1 by GSEA analysis (Figure 3E; NES = −1.35) and on the gene level for fibrillar collagens (Figure 3F; $p < 0.05$). Notch signaling blunts the cytokine response in senescence as SASP factors (Figure 3G; NES = 1.1) and the interferon-gamma response (Figure S3D; NES = 1.48) are differentially regulated between mVenus:EV and mVenus:dnMaml1, as judged by GSEA. Importantly, E2F targets, whose downregulation is one of the hallmarks of senescence, are upregulated in mVenus:dnMaml1 cells compared to mVenus:EV (Figure 3H; p = not significant [n.s.]) (Narita et al., 2003), which offers an explanation for the strong phenotype differences we observed between the two conditions (see Figure 3B).

Notch induces senescence in a juxtacrine manner through cell-to-cell contact. We performed transwell experiments to verify the effect of cell-to-cell contact on the secondary senescence transcriptome. We co-cultured ER:Ras cells with GFP cells (GFP contact; Figure 3I) and GFP cells on their own in the transwell of the same well (GFP no contact). In this setting, GFP no contact cells shared media with ER:Ras cells, where cytokines can be transferred but no cell-to-cell contact is possible. We performed bulk RNA-seq of GFP contact and no contact cells 7 days after tamoxifen induction and confirmed enhanced expression of previously observed marker genes for NIS secondary senescence in GFP contact cells (Figure 3J). In addition, GSEA confirmed enrichment of Notch (NES = 1.59, FDR q = 0.019) and TGFB (NES = 1.87, FDR q = 0.0016) signaling (Figures 3K and S3E) in GFP contact cells. Pathway analysis confirmed significant upregulation of previously described senescence pathways, such as “Senescence and Autophagy in Cancer” and “Matrix Metalloproteases” in GFP contact compared to GFP no contact cells (Figure 3L). Equally, GSEA showed repression of E2F target genes in GFP contact compared to GFP no contact fibroblasts (Figure 3M) except for E2F7, which is known to be upregulated in senescence (Figure 3J) (Aksoy et al., 2012). GSEA analysis suggests that the global differences between GFP contact and no contact cells resemble the differences between mVenus:EV and mVenus:dnMaml1 secondary senescence (Figure S3F).

OIS induction is a multi-step process with an early proliferative phase at days 1–3, followed by a phenotype transition phase at days 3–5, and established senescence from day 7 after RasV12 expression (Young et al., 2009). To compare the impact of the different phases of primary OIS onto secondary senescence, we co-cultured mVenus:EV or mVenus:dnMAML1 cells repeatedly with ER:Ras cells at days 3–6 or at days 7–10 after RasV12 induction (Figure S3G). As expected, ER:Ras cells showed low levels of EdU incorporation in mVenus:dnMAML1 (day 7, $p = 0.01$) or mVenus:EV co-culture (day 7, $p < 0.001$) (Figures S3H and S3I) as a result of primary OIS. Co-culturing mVenus:EV with ER:Ras cells in the phenotype transition phase (days 3–5 after RasV12 induction) lead to a significant reduction in EdU when compared to uninduced co-cultures ($p < 0.001$; Figure S3H), suggesting that secondary senescence was induced by transition-phase primary OIS cells. The transition-phase effect is Notch-dependent because it cannot be induced in mVenus:dnMAML1 cells ($p = 0.12$; Figure S3I). In contrast, by co-culturing mVenus:EV cells with primary OIS cells in established senescence phase (days 7–10 after RasV12 induction), we were unable to detect a reduction in EdU incorporation in mVenus:EV cells compared to uninduced co-cultures ($p = 0.59$; Figure S3H), mirroring results obtained in mVenus:dnMAML1 co-cultures ($p = 0.99$; Figure S3I). From day 4 co-culture, we detected a significant upregulation of Notch1 on the cell surface of mVenus:EV ($p = 0.041$ day 4, $p = 0.038$ day 7; data not shown) and mVenus:dnMaml1 ($p = 0.023$ day 4, $p = 0.046$ day 7; data not shown) cells compared to growing, providing a pathway to NIS induction (Figure S3J).

Figure 3. NIS Mediates Secondary Senescence *In Vitro*

(A) Schematic representation of co-cultures with perturbed Notch signaling.

(B) Bar plot for EdU incorporation in growing (black) or senescent (gray) EV or dnMAML1 cells co-cultured with ER:Ras as proportion of all cells scored. Error bars are displayed as SEM; $F[7,16] = 20.63$, $p < 0.001$, one-way ANOVA with Tukey's test. (n = 3 per condition). Representative images are shown.

(C) Scmap cluster projection of the dnMAML1 and EV 10× scRNA-seq dataset to the GFP co-culture 10× scRNA-seq dataset (see Figure 1H).

(D) tSNE plot of single cells colored by the projection toward the GFP co-culture 10× dataset (see Figure 1H). Pie charts show percentage of cells.

(E) GSEA pre-ranked test for enrichment of Notch signaling in mVenus:EV identified as secondary senescence by scmap.

(F) Heatmap of single-cell data comparing mVenus:EV and mVenus:dnMAML1 for collagens and SASP genes. Red, upregulated and blue, downregulated.

(G) GSEA pre-ranked test for enrichment of SASP genes in mVenus:dnMAML1 identified as secondary senescence by scmap.

(H) GSEA pre-ranked test for enrichment of E2F targets in mVenus:dnMAML1 identified as secondary senescence by scmap.

(I) Schematic representation of transwell co-culture assay of OIS and GFP cells.

(J) Heatmap of significantly differentially expressed genes ($p < 0.05$) between GFP contact and GFP no contact cells.

(K) GSEA pre-ranked analysis for enrichment of Notch signaling in GFP contact cells compared to GFP no contact cells.

(L) Pathway analysis for DE genes between GFP contact/GFP no contact ($p < 0.05$).

(M) GSEA pre-ranked analysis for enrichment of E2F targets in GFP no contact compared to GFP contact cells. Leading edge genes are indicated.

Related to Figure S3 and Table S2.

OPEN ACCESS
CellPress

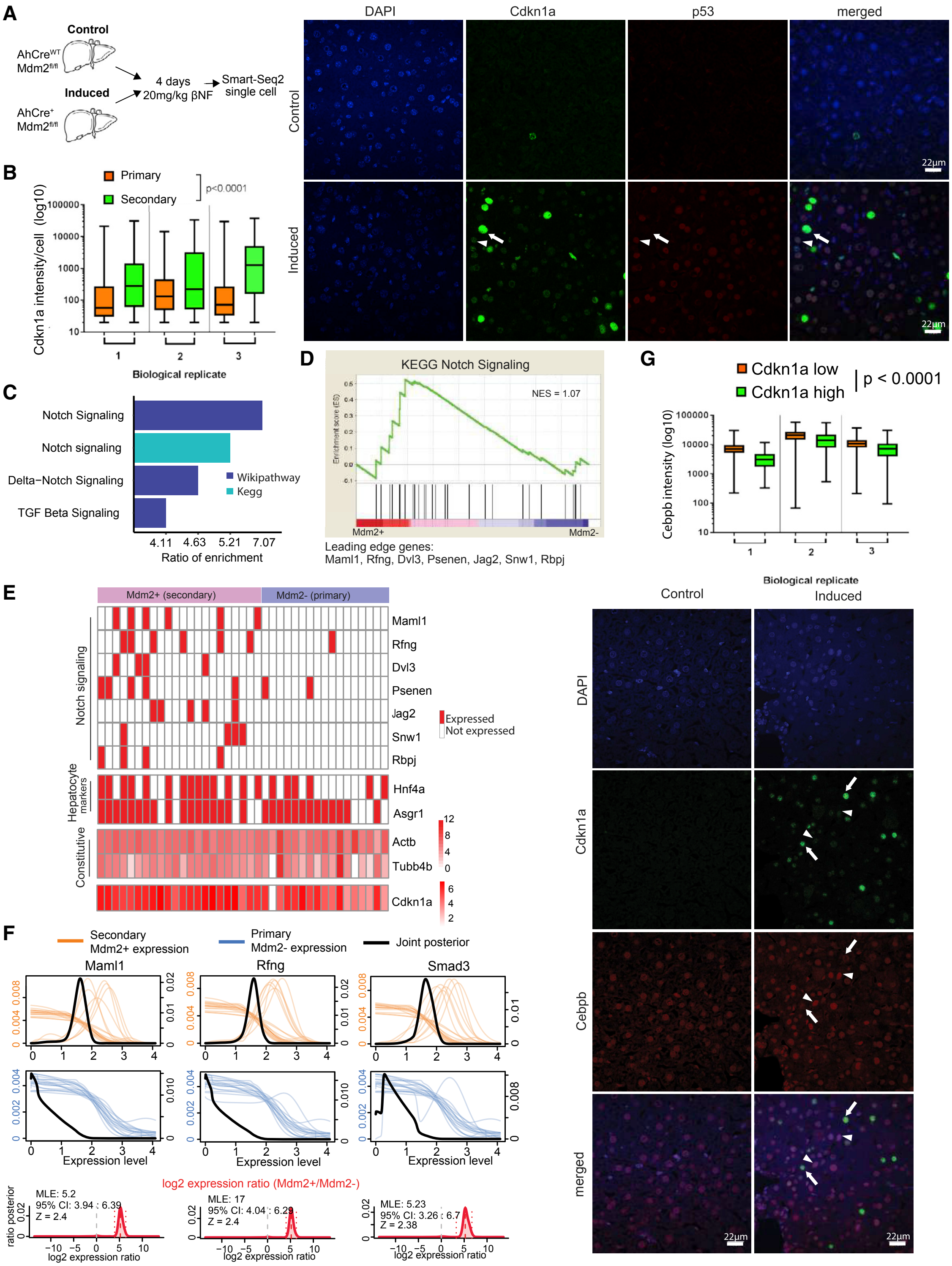

Figure 4. Notch Signaling Mediates Secondary Senescence *In Vivo*

(A) Schematic representation of *in vivo* single-cell experiment.

(B) Representative immunofluorescence images of liver section from induced AhCre+Mdm2fl/fl and control $AhCre^{WT}Mdm2^{fl/fl}$ mice stained for p53 and CDKN1A. Intrinsically induced senescence (arrowhead) and secondary senescence (arrow) are indicated. Boxplot for CDKN1A intensity in primary versus secondary senescent cells. (senescence: $F[1,50291] = 2766$, $p < 0.0001$; biological replicates: $F[2,50291] = 283.2$, $p < 0.0001$; senescence × biological replicates: $F[2,50291] = 280.5$, $p < 0.0001$, two-way ANOVA). Scale bar, 22 μm.

(legend continued on next page)

These results highlight a need for ER:Ras fibroblasts to be in phenotype transition phase to mediate secondary senescence by Notch1. Overall, our data identify Notch as a key mediator of secondary senescence.

Secondary Senescent Hepatocytes Are Characterized by NIS Signature

To test the involvement of NIS *in vivo*, we used a model where primary senescence is induced in a subpopulation of hepatocytes following *Mdm2* deletion (Bird et al., 2018). This model is activated by hepatocyte-targeted recombination of *Mdm2* (β-napthoflavone [βNF] induction AhCre, Mdm2−), resulting in primary senescence in *Mdm2−* cells. *Mdm2−* hepatocytes induce secondary senescence in neighboring hepatocytes (Bird et al., 2018) (Figure 4A). In this model, the presence of p53 induction through *Mdm2* deletion with medium levels of CDKN1A (non-senescence/primary $p < 0.001$) marks primary senescence induction (Bird et al., 2018) (Figure 4B; Figure S4A and S4B). Physiological levels of P53 and high levels of CDKN1A (CDKN1A expression secondary/primary $p < 0.0001$) marks secondary senescence in *Mdm2* normal (*Mdm2+*) hepatocytes as described (Bird et al., 2018) (Figure 4B). Based on these characteristics, cells can be readily distinguished by immunohistochemistry with 23% of primary and 10% of secondary senescence hepatocytes detected (Figure S4A). We have previously shown that both subpopulations of hepatocytes upregulate senescence markers (gH2AX, Il1A, SA-Beta Gal) and reduce BrdU incorporation (Bird et al., 2018).

To establish if primary and secondary senescence can be distinguished based on the transcriptome *in vivo*, we performed scRNA-seq on hepatocytes using Smart-Seq2 (Figure 4A). After filtering (Figure S4B and S4C; Table S1), we retained 39 single cells from induced *Mdm2*-deleted mouse liver for downstream analysis. We distinguished *Mdm2−* cells from *Mdm2+* hepatocytes by the absence of mapping reads over exon 5 and 6 of the *Mdm2* gene (Figure S4D). We detected expression of *Cdkn1a* in both senescent populations consistently with the differences in CDKN1A protein levels detected by immunohistochemistry (Figure 4B), with lower (but not significant) *Cdkn1a* expression in *Mdm2−* compared to *Mdm2+* hepatocytes (Figure S4E), enabling us to distinguish primary and secondary senescence. To verify a senescence phenotype in both *Mdm2−* and *Mdm2+* hepatocyte populations, we conducted pathway analysis with upregulated pathways being enriched in p53 signaling, including CDKN1A, DNA damage response, and cytokine signaling (Figure S4F). We next asked if NIS plays a role in secondary senescence *in vivo* by analyzing our single-cell data by using three independent methods. Differentially expressed genes between *Mdm2+* and *Mdm2−* cells were identified using single cell differential expression (SCDE) (Table S2), and genes were ranked between *Mdm2+*/*Mdm2−* cells for downstream analysis. First, pathway analysis revealed enrichment in Notch signaling (ratio of enrichment [RE], 7.07), Delta-Notch signaling (RE, 4.63), and TGFB (RE, 4.11) signaling pathways (Figure 4C). Second, GSEA revealed Notch signaling pathway (NES = 1.07) as one of the top 20 Kegg pathways enriched in Mdm2+/Mdm2− (Figure 4D) with leading edge genes *Maml1* and *Jag2* detectable mainly in Mdm2+ cells (Fisher's exact test = 6.93×10^{-7}; Figure 4E). Housekeeping and hepatocyte-specific genes were expressed to the same level in the majority of cells regardless of Mdm2 status (Figure 4E). Third, SCDE analysis confirmed the specific upregulation of Notch and TGFB targets *Maml1* (adjusted *Z* score [aZ] = 0.4) and *Rfng* (aZ = 0.39) with effector protein *Smad3* (aZ = 0.26) in *Mdm2+* compared to *Mdm2−* hepatocytes (Figure 4F). To assess the proposed TGFB and CEBPB bias between primary and secondary senescence *in vivo*, we stained livers from uninduced and induced mice for CDKN1A and CEBPB by immunohistochemistry. Consistent with our *in vitro* data, we observed significantly higher CEBPB protein in primary ($p < 0.0001$; Figure 4G) compared to secondary senescent hepatocytes. These lines of evidence show that secondary senescent hepatocytes are characterized by a NIS signature *in vivo*.

DISCUSSION

Cancer heterogeneity is an expanding field of research, with little knowledge about cellular heterogeneity in a pre-cancerous state. Are all cells reacting similarly to oncogene activation or does an oncogenic insult result in a heterogeneous population? Understanding heterogeneity in a pre-cancerous state will inform distinct propensities for transformation in subpopulations. Our study uncovers heterogeneity in primary OIS and secondary senescence transcriptomes following an oncogenic insult using single-cell approaches.

Paracrine induction of senescence is thought to be the main mediator of secondary senescence in OIS (Acosta et al., 2013; Kuilman et al., 2008). Our results challenge this canonical view implicating NIS as a synergistic driver of secondary senescence *in vitro*, in the most studied OIS background (RasV12) and in the liver *in vivo*.

Primary and secondary senescent cells are not thought of as functionally distinct endpoints. We provide strong evidence for differences between primary OIS and Notch-mediated juxtacrine

(C) Pathway analysis for *Mdm2+* (secondary) genes.

(D) GSEA for *Mdm2+*/*Mdm2−* cells (NES = 1.07). Leading edge genes are indicated.

(E) Heatmap for Notch pathway, hepatocyte markers, and *Cdkn1a* genes in *Mdm2+* and *Mdm2−* cells. Constitutive genes and *Cdkn1a* were colored by their expression relative levels (binary: red expressed, white not expressed).

(F) SCDE for *Maml1*, *Rfng*, and *Smad3* in *Mdm2+* cells (orange lines) and *Mdm2−* cells (blue lines). Joint posterior is marked by black line. Fold change of the genes in *Mdm2+*/*Mdm2−* is indicated in red, and dotted lines mark the 95% confidence interval. MLE, maximum likelihood estimation; CI, confidence interval; Z, *Z* score.

(G) Representative immunofluorescence images of liver section from induced and control mice. Primary senescent cells (arrowheads) and secondary senescent cells (arrows) are indicated (CDKN1A: $F[1,60145] = 353.3$, $p < 0.0001$; biological replicates: $F[2,60145] = 1044$, $p < 0.0001$; CDKN1A × biological replicates: $F[2,60145] = 8.96$, $p < 0.0001$, two-way ANOVA). Scale bar, 22 μm.

Related to Figure S4 and Tables S1 and S2.

secondary senescence, as they display distinct gene expression profiles and potentially different transformation potential (Acosta et al., 2013; Hoare et al., 2016). Some of our findings point to a functional diversification, for example, the blunted SASP response and the induction of fibrillar collagens in secondary senescence compared to OIS.

We identified two transcriptional endpoints for primary OIS, namely a Ras-driven and a NIS program. Notch signaling is mediated through cell-to-cell contact (juxtacrine), and Hoare et al. (2016) have shown that it can be a transient state toward primary senescence induction. Our data indicate cells carrying a composite transcriptional signature of paracrine and juxtacrine events as a facultative endpoint for cells with detectable Ras activation (primary). The transformation potential of these heterogeneous populations needs addressing.

STAR★METHODS

Detailed methods are provided in the online version of this paper and include the following:

- KEY RESOURCES TABLE
- CONTACT FOR REAGENT AND RESOURCE SHARING
- EXPERIMENTAL MODEL AND SUBJECT DETAILS
  - Animal models
  - Cell culture
- METHOD DETAILS
  - Hepatocyte isolation
  - Immunohistochemistry
  - Transwell assay
  - Flow cytometry
  - RNA extraction
  - qPCR
  - EdU incorporation and SA-Beta Gal staining
  - Immunofluorescence
  - Confocal microscopy and Image analysis
  - Single cell data generation
- QUANTIFICATION AND STATISTICAL ANALYSIS
  - Bioinformatics analysis
  - Statistical analysis
- DATA AND SOFTWARE AVAILABILITY

SUPPLEMENTAL INFORMATION

Supplemental Information can be found online at https://doi.org/10.1016/j.celrep.2019.03.104.

ACKNOWLEDGMENTS

K.K. was supported by the Wellcome Trust (105641/Z/14/Z). T.C. was supported by a Chancellor's Fellowship held at the University of Edinburgh. N.R. was supported by a PhD studentship funded by the Wellcome Trust Sanger Institute (206194) and the Royal Thai Government. N.O. was supported by MRC (MC_PC_15075). The N.N. lab was partially supported by IDeA grant P20GM109035 (Center for Computational Biology of Human Disease) from NIH NIGMS and grant 1R01AG050582-01A1 from NIH NIA. Y.V.T. was funded by the American Federation for Aging Research. T.G.B. was funded by the Wellcome Trust (WT107492Z). C.K. was supported by CRUK Beatson Institute Core funding. P.D.A. was funded by P01 grant (AG031862) and by CRUK (C10652/A16566). Work in the Green laboratory was supported by Bloodwise, CRUK, and Wellcome Trust. J.-C.A. was supported by CRUK (C47559/A16243). We would like to thank the CRUK Beatson Histology core facility for help with immunohistochemistry and Prof. Chris Ponting and Dr. Andy Fynch for critical reading of the manuscript.

AUTHOR CONTRIBUTIONS

T.C., K.K., and N.N. conceived the study. Y.V.T. performed bioinformatics analysis. N.R. performed the experiments and data analysis with help from A.S., A.Q., N.T., N.O., and K.K. *In vivo* work and data analysis were carried out by C.K., M.M., and T.G.B. P.D.A., J.-C.A., and A.R.G. gave feedback on the study and manuscript. K.K., T.C., and Y.V.T. wrote the manuscript.

DECLARATION OF INTERESTS

The authors declare no competing interests.

Received: September 13, 2018
Revised: March 18, 2019
Accepted: March 27, 2019
Published: April 23, 2019

REFERENCES

Acosta, J.C., O'Loghlen, A., Banito, A., Guijarro, M.V., Augert, A., Raguz, S., Fumagalli, M., Da Costa, M., Brown, C., Popov, N., et al. (2008). Chemokine signaling via the CXCR2 receptor reinforces senescence. Cell *133*, 1006–1018.

Acosta, J.C., Banito, A., Wuestefeld, T., Georgilis, A., Janich, P., Morton, J.P., Athineos, D., Kang, T.W., Lasitschka, F., Andrulis, M., et al. (2013). A complex secretory program orchestrated by the inflammasome controls paracrine senescence. Nat. Cell Biol. *15*, 978–990.

Adams, P.D. (2007). Remodeling of chromatin structure in senescent cells and its potential impact on tumor suppression and aging. Gene *397*, 84–93.

Adams, P.D. (2009). Healing and hurting: molecular mechanisms, functions, and pathologies of cellular senescence. Mol. Cell *36*, 2–14.

Aksoy, O., Chicas, A., Zeng, T., Zhao, Z., McCurrach, M., Wang, X., and Lowe, S.W. (2012). The atypical E2F family member E2F7 couples the p53 and RB pathways during cellular senescence. Genes Dev. *26*, 1546–1557.

Anders, S., Pyl, P.T., and Huber, W. (2015). HTSeq—a Python framework to work with high-throughput sequencing data. Bioinformatics *31*, 166–169.

Bird, T.G., Müller, M., Boulter, L., Vincent, D.F., Ridgway, R.A., Lopez-Guadamillas, E., Lu, W.Y., Jamieson, T., Govaere, O., Campbell, A.D., et al. (2018). TGFβ inhibition restores a regenerative response in acute liver injury by suppressing paracrine senescence. Sci. Transl. Med. *10*, eaan1230.

Braig, M., Lee, S., Loddenkemper, C., Rudolph, C., Peters, A.H., Schlegelberger, B., Stein, H., Dörken, B., Jenuwein, T., and Schmitt, C.A. (2005). Oncogene-induced senescence as an initial barrier in lymphoma development. Nature *436*, 660–665.

Butler, A., Hoffman, P., Smibert, P., Papalexi, E., and Satija, R. (2018). Integrating single-cell transcriptomic data across different conditions, technologies, and species. Nat. Biotechnol. *36*, 411–420.

Campisi, J. (2013). Aging, cellular senescence, and cancer. Annu. Rev. Physiol. *75*, 685–705.

Chandra, T., and Kirschner, K. (2016). Chromosome organisation during ageing and senescence. Curr. Opin. Cell Biol. *40*, 161–167.

Coppé, J.P., Patil, C.K., Rodier, F., Sun, Y., Muñoz, D.P., Goldstein, J., Nelson, P.S., Desprez, P.Y., and Campisi, J. (2008). Senescence-associated secretory phenotypes reveal cell-nonautonomous functions of oncogenic RAS and the p53 tumor suppressor. PLoS Biol. *6*, 2853–2868.

Criscione, S.W., Teo, Y.V., and Neretti, N. (2016). The Chromatin Landscape of Cellular Senescence. Trends Genet. *32*, 751–761.

Di Micco, R., Fumagalli, M., Cicalese, A., Piccinin, S., Gasparini, P., Luise, C., Schurra, C., Garre', M., Nuciforo, P.G., Bensimon, A., et al. (2006).

Oncogene-induced senescence is a DNA damage response triggered by DNA hyper-replication. Nature *444*, 638–642.

Garrison, E., and Marth, G. (2012). Haplotype-based variant detection from short-read sequencing. arXiv, arXiv:1207.3907. https://arxiv.org/abs/1207.3907.

Hoare, M., Ito, Y., Kang, T.W., Weekes, M.P., Matheson, N.J., Patten, D.A., Shetty, S., Parry, A.J., Menon, S., Salama, R., et al. (2016). NOTCH1 mediates a switch between two distinct secretomes during senescence. Nat. Cell Biol. *18*, 979–992.

Jun, J.I., and Lau, L.F. (2010). Cellular senescence controls fibrosis in wound healing. Aging (Albany N.Y.) *2*, 627–631.

Kharchenko, P.V., Silberstein, L., and Scadden, D.T. (2014). Bayesian approach to single-cell differential expression analysis. Nat. Methods *11*, 740–742.

Kim, D., Langmead, B., and Salzberg, S.L. (2015). HISAT: a fast spliced aligner with low memory requirements. Nat. Methods *12*, 357–360.

Kirschner, K., Samarajiwa, S.A., Cairns, J.M., Menon, S., Pérez-Mancera, P.A., Tomimatsu, K., Bermejo-Rodriguez, C., Ito, Y., Chandra, T., Narita, M., et al. (2015). Phenotype specific analyses reveal distinct regulatory mechanism for chronically activated p53. PLoS Genet. *11*, e1005053.

Kirschner, K., Chandra, T., Kiselev, V., Flores-Santa Cruz, D., Macaulay, I.C., Park, H.J., Li, J., Kent, D.G., Kumar, R., Pask, D.C., et al. (2017). Proliferation Drives Aging-Related Functional Decline in a Subpopulation of the Hematopoietic Stem Cell Compartment. Cell Rep. *19*, 1503–1511.

Kiselev, V.Y., Kirschner, K., Schaub, M.T., Andrews, T., Yiu, A., Chandra, T., Natarajan, K.N., Reik, W., Barahona, M., Green, A.R., and Hemberg, M. (2017). SC3: consensus clustering of single-cell RNA-seq data. Nat. Methods *14*, 483–486.

Kiselev, V.Y., Yiu, A., and Hemberg, M. (2018). scmap: projection of single-cell RNA-seq data across data sets. Nat. Methods *15*, 359–362.

Kuilman, T., Michaloglou, C., Vredeveld, L.C., Douma, S., van Doorn, R., Desmet, C.J., Aarden, L.A., Mooi, W.J., and Peeper, D.S. (2008). Oncogene-induced senescence relayed by an interleukin-dependent inflammatory network. Cell *133*, 1019–1031.

Li, H. (2011). A statistical framework for SNP calling, mutation discovery, association mapping and population genetical parameter estimation from sequencing data. Bioinformatics *27*, 2987–2993.

Love, M.I., Huber, W., and Anders, S. (2014). Moderated estimation of fold change and dispersion for RNA-seq data with DESeq2. Genome Biol. *15*, 550.

Lu, W.Y., Bird, T.G., Boulter, L., Tsuchiya, A., Cole, A.M., Hay, T., Guest, R.V., Wojtacha, D., Man, T.Y., Mackinnon, A., et al. (2015). Hepatic progenitor cells of biliary origin with liver repopulation capacity. Nat. Cell Biol. *17*, 971–983.

Lun, A.T., McCarthy, D.J., and Marioni, J.C. (2016). A step-by-step workflow for low-level analysis of single-cell RNA-seq data with Bioconductor. F1000Res. *5*, 2122.

McCarthy, D.J., Campbell, K.R., Lun, A.T., and Wills, Q.F. (2017). Scater: pre-processing, quality control, normalization and visualization of single-cell RNA-seq data in R. Bioinformatics *33*, 1179–1186.

Mosteiro, L., Pantoja, C., Alcazar, N., Marión, R.M., Chondronasiou, D., Rovira, M., Fernandez-Marcos, P.J., Muñoz-Martin, M., Blanco-Aparicio, C., Pastor, J., et al. (2016). Tissue damage and senescence provide critical signals for cellular reprogramming in vivo. Science *354*, aaf4445.

Narita, M., Nũnez, S., Heard, E., Narita, M., Lin, A.W., Hearn, S.A., Spector, D.L., Hannon, G.J., and Lowe, S.W. (2003). Rb-mediated heterochromatin formation and silencing of E2F target genes during cellular senescence. Cell *113*, 703–716.

Nelson, G., Wordsworth, J., Wang, C., Jurk, D., Lawless, C., Martin-Ruiz, C., and von Zglinicki, T. (2012). A senescent cell bystander effect: senescence-induced senescence. Aging Cell *11*, 345–349.

Osorio, F.G., Bárcena, C., Soria-Valles, C., Ramsay, A.J., de Carlos, F., Cobo, J., Fueyo, A., Freije, J.M., and López-Otín, C. (2012). Nuclear lamina defects cause ATM-dependent NF-κB activation and link accelerated aging to a systemic inflammatory response. Genes Dev. *26*, 2311–2324.

Parry, A.J., Hoare, M., Bihary, D., Hänsel-Hertsch, R., Smith, S., Tomimatsu, K., Mannion, E., Smith, A., D'Santos, P., Russell, I.A., et al. (2018). NOTCH-mediated non-cell autonomous regulation of chromatin structure during senescence. Nat. Commun. *9*, 1840.

Picelli, S., Faridani, O.R., Björklund, A.K., Winberg, G., Sagasser, S., and Sandberg, R. (2014). Full-length RNA-seq from single cells using Smart-seq2. Nat. Protoc. *9*, 171–181.

Qiu, X., Mao, Q., Tang, Y., Wang, L., Chawla, R., Pliner, H.A., and Trapnell, C. (2017). Reversed graph embedding resolves complex single-cell trajectories. Nat. Methods *14*, 979–982.

Ritchie, M.E., Phipson, B., Wu, D., Hu, Y., Law, C.W., Shi, W., and Smyth, G.K. (2015). limma powers differential expression analyses for RNA-sequencing and microarray studies. Nucleic Acids Res. *43*, e47.

Ritschka, B., Storer, M., Mas, A., Heinzmann, F., Ortells, M.C., Morton, J.P., Sansom, O.J., Zender, L., and Keyes, W.M. (2017). The senescence-associated secretory phenotype induces cellular plasticity and tissue regeneration. Genes Dev. *31*, 172–183.

Rousseeuw, P. (1987). Silhouettes: A Graphical Aid to the Interpretation and Validation of Cluster Analysis. J. Comput. Appl. Math. *20*, 53–65.

Serrano, M., Lin, A.W., McCurrach, M.E., Beach, D., and Lowe, S.W. (1997). Oncogenic ras provokes premature cell senescence associated with accumulation of p53 and p16INK4a. Cell *88*, 593–602.

Soria-Valles, C., Osorio, F.G., Gutiérrez-Fernández, A., De Los Angeles, A., Bueno, C., Menéndez, P., Martín-Subero, J.I., Daley, G.Q., Freije, J.M., and López-Otín, C. (2019). Retraction. Nat. Cell Biol. *21*, 410.

Subramanian, A., Tamayo, P., Mootha, V.K., Mukherjee, S., Ebert, B.L., Gillette, M.A., Paulovich, A., Pomeroy, S.L., Golub, T.R., Lander, E.S., and Mesirov, J.P. (2005). Gene set enrichment analysis: a knowledge-based approach for interpreting genome-wide expression profiles. Proc. Natl. Acad. Sci. USA *102*, 15545–15550.

van Deursen, J.M. (2014). The role of senescent cells in ageing. Nature *509*, 439–446.

Wang, J., Vasaikar, S., Shi, Z., Greer, M., and Zhang, B. (2017). WebGestalt 2017: a more comprehensive, powerful, flexible and interactive gene set enrichment analysis toolkit. Nucleic Acids Res. *45*, W130–W137.

Watanabe, S., Kawamoto, S., Ohtani, N., and Hara, E. (2017). Impact of senescence-associated secretory phenotype and its potential as a therapeutic target for senescence-associated diseases. Cancer Sci. *108*, 563–569.

Witten, D.M., and Tibshirani, R. (2010). A framework for feature selection in clustering. J. Am. Stat. Assoc. *105*, 713–726.

Xue, W., Zender, L., Miething, C., Dickins, R.A., Hernando, E., Krizhanovsky, V., Cordon-Cardo, C., and Lowe, S.W. (2007). Senescence and tumour clearance is triggered by p53 restoration in murine liver carcinomas. Nature *445*, 656–660.

Young, A.R., Narita, M., Ferreira, M., Kirschner, K., Sadaie, M., Darot, J.F., Tavaré, S., Arakawa, S., Shimizu, S., Watt, F.M., and Narita, M. (2009). Autophagy mediates the mitotic senescence transition. Genes Dev. *23*, 798–803.

STAR★METHODS

KEY RESOURCES TABLE

REAGENT or RESOURCE	SOURCE	IDENTIFIER
Antibodies		
Rat monoclonal anti-p21 (Cdkn1a)	Originally gift from Serrano lab CNIO, Madrid, now available at Abcam	HUGO291-T3413
C/EBPB clone 1H7	Abcam	Cat# ab15050; RRID:AB_301598
NCL-L-p53-CM5p	Leica Biosystems	Cat# P53-CM5P-L; RRID:AB_2744683
Notch1-PE FAB5317P	R&D systems	Cat# FAB5317P-025; RRID:AB_1602927
Mouse monoclonal anti-p53	Cell Signaling Technology	Cat#2524; RRID:AB_331743
VECTASTAIN® Elite® ABC HRP Reagent	Vector Labs	PK-7100; RRID:AB_2336827
TSA Plus Fluorescein Evaluation Kit - FITC	Perkin Elmer	NEL741B001KT
TSA Plus Fluorescein Evaluation Kit – Cy3	Perkin Elmer	NEL744B001KT
Biotinylated polyclonal goat anti rat	Vector Labs	BA-9400; RRID:AB_2336202
Biotinylated polyclonal horse anti mouse	Vector Labs	BA-2000; RRID:AB_2313581
C/EBPB clone E299	Abcam	Cat# ab 32358; RRID:AB_726796
Chemicals, Peptides, and Recombinant Proteins		
4-hydroxytamoxifen	Sigma	Cat# H6278
7-AAD	Biolegend	Cat# 420403
DAPI	Biolegend	Cat# 422801
β-Naphthoflavone	Sigma	Cat# N3633
Critical Commercial Assays		
Tetro cDNA synthesis kit	Bioline	Cat# BIO-65043
Click-iT EdU Alexa Fluor 555 imaging kit	Click-iT EdU Alexa Fluor 555 imaging kit	Cat# 32727
RNeasy Mini Kit	QIAGEN	Cat# 74104
Chromium Single Cell 3′ Library & Gel Bead Kit v2	10xGenmics	Cat# 120237
Chromium i7 Multiplex Kit	10xGenomics	Cat# 120262
Chromium Single Cell 3′ Chip Kit v2	10xGenomics	Cat# 120236
Deposited Data		
All single cell RNA-seq datasets	This paper	GEO: GSE115301
Bulk RNA-seq data	This paper	GEO: GSE115301
Raw Imaging data	This paper	Mendeley dataset https://doi.org/10.17632/y76pb7s8h3.1.
Experimental Models: Cell Lines		
IMR90 normal human diploid fibroblasts	ATTC	ATCC Cat# CRL-7931, RRID:CVCL_0347
Experimental Models: Organisms/Strains		
Mouce: AhCre+, Mdm2$^{flox/flox}$	Bird et. al. 2018	N/A
Oligonucleotides		
Primers for qPCR, see Table S3	This paper	N/A
Recombinant DNA		
pLNCX2 ER:ras	Addgene, Young et al., 2009	Plasmid #67844
pLPC-puro dnMAML1-mVenus	a kind gift from M. Narita to J.C.A.	Hoare et al. 2016
pLPC-puro -mVenus	a kind gift from M. Narita to J.C.A.	Hoare et al. 2016
pGIPZ-GFP	a kind gift from M. Narita to J.C.A.	Hoare et al. 2016
Software and Algorithms		
TrimGalore	Babraham Institute	http://www.bioinformatics.babraham.ac.uk/projects/trim_galore/ RRID:SCR_016946

(Continued on next page)

Continued		
REAGENT or RESOURCE	SOURCE	IDENTIFIER
HTSeq-0.6.1	Anders et al., 2015	https://htseq.readthedocs.io/en/release_0.11.1/overview.html RRID:SCR_005514
Cell Ranger 2.0.1	10xGenomics	https://support.10xgenomics.com/single-cell-gene-expression/software/pipelines/latest/what-is-cell-ranger
FreeBayes v0.9.20-8-gfef284a	Garrison and Marth, 2012	https://github.com/ekg/freebayes RRID:SCR_010761
HISAT v2.0.1beta	Kim et al., 2015	http://ccb.jhu.edu/software/hisat2/index.shtml RRID:SCR_015530
Monocle2	Qiu et al., 2017	http://cole-trapnell-lab.github.io/monocle-release/docs/ RRID:SCR_016339
SC3	Kiselev et al., 2017	http://bioconductor.org/packages/release/bioc/html/SC3.html RRID:SCR_015953
WebGestalt	Wang et al., 2017	http://www.webgestalt.org/ RRID:SCR_006786
Gene Set Enrichment Analysis	Subramanian et al., 2005	http://software.broadinstitute.org/gsea/index.jsp RRID:SCR_003199
DESeq2	Love et al., 2014	https://bioconductor.org/packages/release/bioc/html/DESeq2.html RRID:SCR_015687
samtools/1.2 mpileup	Li 2011	http://www.htslib.org/ RRID:SCR_002105
SCDE v1.99.1	Kharchenko et al., 2014	http://hms-dbmi.github.io/scde/index.html RRID:SCR_015952
Seurat 2.3.0		http://seurat.r-forge.r-project.org/ RRID:SCR_007322
sparcl 1.0.3	Witten and Tibshirani 2010	https://cran.r-project.org/web/packages/sparcl/index.html

CONTACT FOR REAGENT AND RESOURCE SHARING

Further information and requests for resources and reagents should be directed to and will be fulfilled by the Lead Contact, Tamir Chandra (tamir.chandra@igmm.ed.ac.uk).

EXPERIMENTAL MODEL AND SUBJECT DETAILS

Animal models

Animal welfare conditions have been previously described (Lu et al., 2015). All animal experiments were carried out on healthy, treatment naive animals under procedural guidelines, severity protocols and within the UK with ethical permission from the Animal Welfare and Ethical Review Body (AWERB) and the Home Office (UK). AhCre+/WT *Mdm2*fl/fl and AhCreWT/WT *Mdm2*fl/fl mice (colony N4 C57/Bl6J background) were crossed. Male littermates were housed together, and when used in experiments were all > 20 g body weight and of 10-16 weeks age. Genotyping and single i.p. injection of β-Naphthoflavone (βNF, Sigma UK) at 20mg/kg were performed as previously described (Bird et al., 2018).

Cell culture

We used normal diploid human female lung fibroblasts IMR90 isolated at 16 weeks of gestation for all *in vitro* assays (ATCC® CCL-186). pLNCX2-ER:ras^{G12V}-expressing IMR90 (plasmid obtained from Addgene #67844) were maintained and senescence induced as described under 5% O_2 conditions (Young et al., 2009). ER:IMR90 cells were co-cultured with IMR90:GFP (pGIPZ-GFP, a kind gift from M. Narita to J.C.A.) or an empty vector fused with mVenus (pLPC-puro-mVenus, a kind gift from M. Narita to J.C.A.) or with a dominant negative form of MAML1 fused with mVenus (pLPC-puro-dnMAML1-mVenus, a kind gift from M. Narita to J.C.A.) cells at 10:1 ratio.

METHOD DETAILS

Hepatocyte isolation

Ex vivo primary hepatocytes were isolated using a modified retrograde perfusion technique as previously described (Lu et al., 2015). Hepatocytes were purified by pelleting through a 40% (v:v) percoll gradient prior to FACS sorting.

Immunohistochemistry

Mouse livers were harvested and partially stored in paraffin blocks following fixation in 10% formalin (in PBS) for 18 hours prior to embedding. Immunohistochemistry was performed as described (Bird et al., 2018). Three μm thick paraffin sections were double stained for p53/CDKN1A and CDKN1A and CEBPB using the CDKN1A clone HUGO291H (a gift from Serrano lab, CNIO in Madrid), and either C/EBPB clone 1H7 (Abcam) or p53 clone 1C12 (Cell Signaling). Detection was performed with TSA-Cy3 (Perkin Elmer, NEL744B001KT, 1:50) and TSA-FITC (Perkin Elmer, NEL741B001KT, 1:50). Images were captured on a Zeiss 710 Upright Confocal Z6008 microscope. Stained slides were scanned using the Opera Phoenix High Content screening system (Perkin Elmer) scanner and analyzed using the Columbus software.

Transwell assay

ER:Ras^{G12V}-expressing cells were co-cultured with IMR90:GFP cells. The co-cultured cells were placed into the lower chamber of a transwell system (density $5x10^3$ cells/well). Another pure population of IMR90:GFP cells were cultured in the upper chamber of the transwell system. All cells were maintained in 4-hydroxytamoxifen (Sigma) for 7 days. All experiments were performed in triplicate.

Flow cytometry

Flow cytometry was performed with three independent replicates as previously described (Kirschner et al., 2017) using 7-AAD (Biolegend), DAPI (Biolegend) and anti-Notch1-PE (R&D systems, FAB5317P, 1:20). Analysis was performed on the BD FACSAria II (BD Biosciences, San Jose, CA) using the BD CellQuest PRO software (BD Biosciences, San Jose, CA). Flow data were analyzed with FlowJo v10 (Tree Star, Ashland, OR).

RNA extraction

RNA from three to four independent experiments was extracted using the RNeasy Mini Kit (QIAGEN). All RNA passed with a RIN of 9 or above as determined by Bioanalyser profiling. Ribosome depletion was performed prior to bulk RNA sequencing.

qPCR

cDNA was generated as previously described using the Tetro cDNA synthesis kit (Bioline) (Kirschner et al., 2015). qPCR was performed on a LightCycler 480 (Roche) using Sybr Green method as previously described (Kirschner et al., 2015). Primer sequences are in Table S3.

EdU incorporation and SA-Beta Gal staining

EdU incorporation and SA-Beta gal staining was performed as previously described (Kirschner et al., 2015). EdU incorporation was detected using the Click-iT EdU Alexa Fluor 555 imaging kit (ThermoScientific). For stable cell cycle arrest, cells were co-cultured for two weeks, separated by FACS according to GFP status and cultured as OIS and GFP cells for another week before pulsing them with EdU for 24 hours.

Immunofluorescence

Immunoflurorescence was performed as previously described (Kirschner et al., 2015). Anti- C/EBPB clone E299 (Abcam) was used as 1:500 dilution.

Confocal microscopy and Image analysis

BriteMac confocal microscope was used to visualize cells at 40x. Images were analyzed using ImageJ. Percentages of SAHF, YFP/GFP and EdU-positive cells were calculated by assessing 1600-2000 cells per experiment from three independent experiments.

Single cell data generation

Smart-Seq2 was performed on sorted ER:IMR90 cells or hepatocytes as previously described (Kirschner et al., 2017). Single cell data for all co-culture experiments were generated using the Chromium Single Cell 3′ Chip Kit v2 (10xGenomics), following the manufacturer's instructions.

QUANTIFICATION AND STATISTICAL ANALYSIS

Bioinformatics analysis

Sequencing reads processing, alignment and quantification of time-course experiment

Smart-Seq2 generated paired-end reads were quality trimmed using Trim galore (http://www.bioinformatics.babraham.ac.uk/projects/trim_galore/) and aligned to the human reference genome, hg19, neomycin sequence from pLNCX2-ER-ras_neo, ERCC spike-in sequences and RasV12 using HISAT v2.0.1beta (Kim et al., 2015). Cells with less than 200,000 hg19 aligned reads, and a ratio of ERCC RNA spike-in control aligned reads to total aligned reads greater than 0.5 were omitted. hg19 aligned reads were randomly downsampled to 200,000 reads. Genes were quantified using HTSeq-0.6.1 (Anders et al., 2015). Cells with more than 80,000 total gene counts and at least 500 genes with at least one count were used for downstream analysis. 224 IMR90 cells

(100 Growing cells, 41 Day 2 cells, 42 Day 4 cells and 41 senescent cells) passed this second filtering step and used for downstream analyses.

Sequencing reads processing, alignment, quantification and analysis of 10x Chromium RNA-seq data

Cell Ranger 2.0.1 (10x Genomics) was used to align the GFP and ER:Ras^{G12V} co-culture 10x Chromium RNA-seq reads to hg19, TurboGFP, puromycin sequence from pGIPZ and neomycin sequence from pLNCX2-ER-ras_neo, and to generate gene-cell matrices. The growing and senescence dataset were aggregated using "cellranger aggr." The data were subsequently processed using Seurat 2.3.0 with cells with less than 15% mitochondrial reads and at least 2500 number of genes being retained (Butler et al., 2018). Seurat 2.3.0 with the default parameters (unless otherwise stated) was used to generate the t-SNE plots (resolution:0.4; dimensions used: 1:15) and three clusters were identified using sparcl 1.0.3 (https://cran.r-project.org/web/packages/sparcl/index.html). SCDE v1.99.1 was used to identify differentially expressed genes between OIS cluster and secondary senescent cluster (Kharchenko et al., 2014). The DE genes (p values < 0.05) (Table S2) were used as the defined gene sets for GSEA Preranked analysis of NIS and RIS log2FC ranked genes. GFP+ cells were identified as cells with > 0.3 normalized expression of GFP or puromycin and Ras+ cells were identified as cells with non-zero expression of neomycin or one or more reads supporting the G > T mutation at Chr11:534288 as identified by FreeBayes v0.9.20-8-gfef284a (Garrison and Marth, 2012). Integration analysis between Smart-seq2 time-point data and 10x data were performed using the canonical correlation analysis in Seurat 2.3.0, in which the union of the top 50 highest dispersion genes and the first two dimensions were used.

Cell Ranger 2.0.1 (10x Genomics) was used to align the 10x Chromium RNA-seq reads from mVenus:dnMAML1 or mVenus:EV co-cultured with ER:Ras^{G12V} cells to hg19, mVenus sequence, puromycin sequence from pLPC-puro and neomycin sequence from pLNCX2-ER-ras_neo to generate gene-cell matrices. mVenus cells were identified as cells with more than zero normalized expression of mVenus or puromycin and Ras+ cells were identified as cells with non-zero expression of neomycin or one or more reads supporting the G > T mutation at Chr11:534288 as identified by FreeBayes v0.9.20-8-gfef284a (Garrison and Marth, 2012). The data were subsequently processed using Seurat 2.3.0 with cells with less than 10% mitochondrial reads and at least 2500 genes being retained. Seurat 2.3.0 with the default parameters (unless otherwise stated) was used to generate the tSNE plots (resolution:0.6; dimensions used: 1:7). The cells were projected to the 10x Chromium GFP and ER:Ras^{G12V} co-culture dataset using scmap-cluster v1.4.1.

Sequencing reads processing, alignment and quantification of in vivo *data*

Smart-Seq2 generated paired-end reads were quality trimmed using Trim galore (http://www.bioinformatics.babraham.ac.uk/projects/trim_galore/) and aligned to the mouse reference genome mm10 and ERCC spike-in sequences using HISAT v2.0.1beta (Kim et al., 2015). The mm10 aligned reads were randomly downsampled to 50,000 reads. Cells with less than 50,000 reads, less than 20,000 gene count, less than 500 genes with at least one read detected and with the log-transformed number of expressed genes and library size of 3 median absolute deviation below the median value were removed (Lun et al., 2016). 39 single cells from the induced hepatocytes and 19 cells from the uninduced hepatocytes passed these filters. 22 primary senescent cells were identified from the induced hepatocytes as cells with no reads mapping over exon 5 and 6 (chr10:117695953-117696049, chr10:117696381-117696439, chr10:117701565-117701614 and chr10:117702202-117702335) of *Mdm2* gene before the downsampling. 17 cells were classified as secondary hepatocytes as judged by their gene expression profiles. Differential genes expression between *Mdm2*+ cells and *Mdm2*- cells was identified using SCDE v1.99 and log2FC ranked gene list from SCDE was used in GSEA pre-ranked analysis. Genes with more than zero log-transformed normalized count (McCarthy et al., 2017) were labeled red, and otherwise white in the binary heatmap. Pathway enrichment was identified using WebGestalt (Wang et al., 2017) with genes that have a z-score of greater than 2 in *Mdm2*+ cells /*Mdm2*- comparison.

Differential gene expression analysis and temporal ordering of cells

We used raw counts from HTSeq-0.6.1 (Anders et al., 2015) as an input to single-cell differential expression (SCDE v1.99.1) (Kharchenko et al., 2014) for differential gene expression analysis between growing and senescence. Cut-off for significantly differentially expressed (DE) was set at 0.05. The expression magnitude (fragments per million) was obtained from SCDE and converted to FPKM as an input for Monocle2 (Qiu et al., 2017). Monocle2 was used to order the transitions of senescent cells of different time points at a pseudo-temporal resolution, and single-cell data were reduced to a 2-dimensional space by using the DDRTree algorithm implemented in Monocle2 (Qiu et al., 2017). Specifically, DE genes between senescence and growing conditions that were identified in SCDE were used to define the trajectory. A consensus clustering approach, SC3, was also applied to the raw count of single cells and used to cluster senescent cells (Kiselev et al., 2017).

Detection of Ras^{V12} construct in Smart-seq2 dataset

We counted reads with a G > T mutation at Chr11:534288 using samtools v1.2 mpileup and bcftools v1.2 (Li, 2011). Cells with more than 1 read supporting over G > T mutation or at least 9 reads mapping to the neomycin sequence are considered as Ras^{V12} positive cells.

Paracrine-induced senescence and RIS microarray data analysis

Log2 RMA signal intensity of RIS IMR90 cells and IMR90 co-cultured in transwells with RIS cells were obtained from GEO GSE41318. Differentially expressed genes were identified using limma (Ritchie et al., 2015) and an adjusted p value of 0.05 was used as the cut-off for significant genes.

Notch and Ras-induced senescence data and GSEA analysis

We used NIS and RIS RNA-seq data with accession number GSE72404. Reads were aligned to as described above. Differential gene expression analysis between NIS and RIS was performed using DESeq2 (Love et al., 2014). The log2 fold change for each gene was used to rank the list of genes in GSEA Preranked analysis (Subramanian et al., 2005). Differentially expressed (DE) genes between senescence top and bottom were identified using SCDE with a p value cutoff of 0.05. The DE genes defined the gene set in GSEA Preranked analysis.

Sequencing reads alignment and quantification of transwell bulk RNA-sequencing data

Reads were aligned to the human reference genome hg19 using HISAT v2.0.1beta (Kim et al., 2015) and those that mapped to annotated genes were quantified using HTSeq-0.6.1 (Anders et al., 2015). Differential gene expression was determined using DESeq2 v1.22.1 (Love et al., 2014). Over-representation analysis was performed using WebGestalt (Wang et al., 2017) and GSEA pre-ranked analysis was performed using the ranking of genes based on the log2FC between GFP contact and GFP no contact.

Statistical analysis

All t tests and one-way ANOVA for the *in vitro* data were performed in R. TukeyHSD was used as the post hoc test for one-way ANOVA. For the *in vitro* data, each experiment and measurement were obtained from three independent experiments unless otherwise specified in the figure legends. Barplots are represented as means with SEM. Statistical significance was set at $p < 0.05$. t test for the *in vivo* data was performed in R and the two-way ANOVA followed by Tukey's test for the *in vivo* data was performed using GraphPad Prism. All animal data were obtained from three biological replicates. Details of all statistical analysis can be found in associated figure legends. For qPCR analysis, Delta delta Ct method was used for quantification with error bars resulting from the delta Ct expression of three to four biological replicates. A two-sided t test was used to calculate p values.

DATA AND SOFTWARE AVAILABILITY

All scRNA-seq and bulk RNA-seq experiments are accessible through GEO accession number GEO: GSE115301. All imaging data are available as Mendeley dataset under https://doi.org/10.17632/y76pb7s8h3.1.

Cell Reports, Volume 27

Supplemental Information

Notch Signaling Mediates Secondary Senescence

Yee Voan Teo, Nattaphong Rattanavirotkul, Nelly Olova, Angela Salzano, Andrea Quintanilla, Nuria Tarrats, Christos Kiourtis, Miryam Müller, Anthony R. Green, Peter D. Adams, Juan-Carlos Acosta, Thomas G. Bird, Kristina Kirschner, Nicola Neretti, and Tamir Chandra

Figure S1

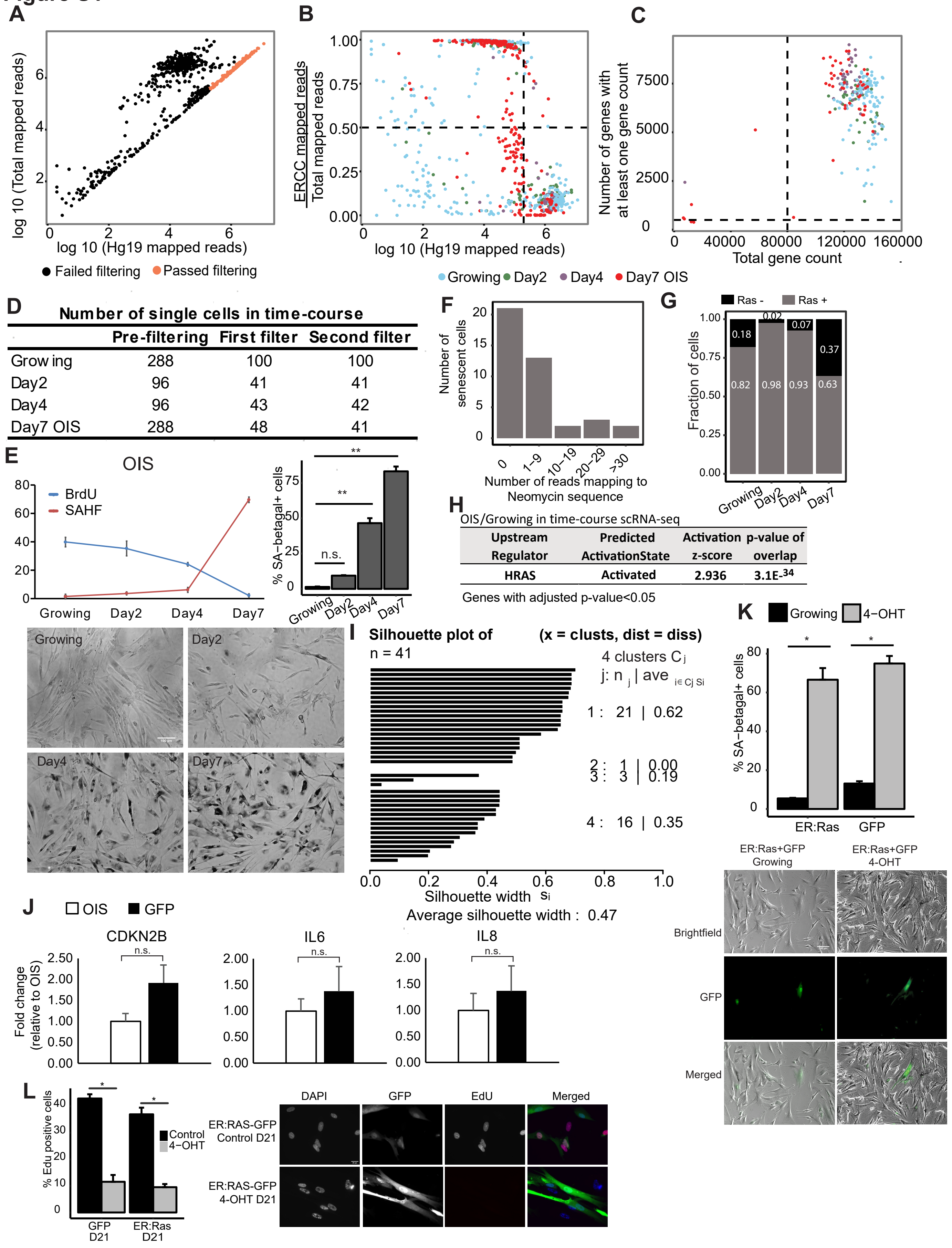
A
log 10 (Total mapped reads)
log 10 (Hg19 mapped reads)
Failed filtering
Passed filtering
B
ERCC mapped reads / Total mapped reads
log 10 (Hg19 mapped reads)
C
Number of genes with at least one gene count
Total gene count
Growing
Day2
Day4
Day7 OIS
D
Number of single cells in time-course
Pre-filtering First filter Second filter
Growing 288 100 100
Day2 96 41 41
Day4 96 43 42
Day7 OIS 288 48 41
E
OIS
BrdU
SAHF
Growing Day2 Day4 Day7
% SA-betagal+ cells
n.s.
**
**
Growing Day2 Day4 Day7
F
Number of senescent cells
0 1-9 10-19 20-29 >30
Number of reads mapping to Neomycin sequence
G
Ras -
Ras +
Fraction of cells
0.18 0.82
0.02 0.98
0.07 0.93
0.37 0.63
Growing Day2 Day4 Day7
H
OIS/Growing in time-course scRNA-seq
Upstream Regulator | Predicted ActivationState | Activation z-score | p-value of overlap
HRAS | Activated | 2.936 | 3.1E-34
Genes with adjusted p-value<0.05
I
Silhouette plot of (x = clusts, dist = diss)
n = 41
4 clusters Cj
j: nj | ave i∈Cj Si
1 : 21 | 0.62
2 : 1 | 0.00
3 : 3 | 0.19
4 : 16 | 0.35
Silhouette width Si
Average silhouette width : 0.47
Growing
Day2
Day4
Day7
J
OIS
GFP
CDKN2B
IL6
IL8
n.s.
Fold change (relative to OIS)
K
Growing
4-OHT
% SA-betagal+ cells
ER:Ras
GFP
ER:Ras+GFP Growing
ER:Ras+GFP 4-OHT
Brightfield
GFP
Merged
L
% Edu positive cells
Control
4-OHT
GFP D21
ER:Ras D21
DAPI
GFP
EdU
Merged
ER:RAS-GFP Control D21
ER:RAS-GFP 4-OHT D21

Figure S1, Related to Figure 1.

(A,B) Filtering according to total mapped reads. Cells with less than 200,000 human aligned reads and with a ratio of ERCC RNA spike-in control aligned reads to total aligned reads that is greater than 0.5 were removed.

(C) The second filtering step was performed to retain cells that have greater than 80,000 total gene counts and at least 500 genes with at least one count. Cell were normalised by downsampling to 200,000 aligned reads for downstream analysis.

(D) The number of cells that passed the filtering step in (A) and (B)

(E) BrdU, SAHF and SA-Beta galactosidase counts in ER:Ras fibroblasts. Days indicated time of tamoxifen treatment. Error bars are SEM, n=3 for each time point. $F[3,8] = 234.8$, $p<0.001$; **$p < 0.001$ using one-way ANOVA with Tukey's test. Scale bar 100µm.

(F) Number of reads aligning to the neomycin sequence from the pLNCX2-ER-ras_neo construct in senescent single cells in the time-course experiment.

(G) Fraction of cells that are RasV12+ in each condition in the time-course experiment.

(H) IPA analysis of OIS/growing in time-course scRNA-seq.

(I) Silhouette plot to assess the quality of clustering. The average silhouette width was 0.47.

(J) Box plots for gene expression of *CDKN2B* (n=3), *IL6* (n=3), and *IL8* (n=3) mRNA measured by qPCR in OIS and GFP cells. Unpaired Student's t-test showed no significant difference in senescent markers expression between OIS and GFP cells. Error bars represent SEM.

(K) SA-Beta galactosidase counts in OIS and GFP cell s. (OIS t = 10.199, df = 2.0096, p= 0.009; GFP t = 15.239, df = 2.3673, p= 0.002 using unpaired Student's t-test). Scale bar 100µm.

(L) Bar plots showing EdU incorporation in GFP cells co-cultured with ER:Ras cells after 21 days as proportion of all cells scored. Error bars are displayed as SEM; **$p<0.001$, *$p<0.05$. Representative images are shown. Scale bar 20µm. (ER:Ras t = -9.899, df = 2.86 68, p= 0.0024; GFP t = 10.395, df = 3.3348, p= 0.0012 using unpaired Student's t-test) (n=3 per experiment).

Figure S2

A **Top activated and shared upstream regulator assessed by IPA**

Time-course
(Secondary senescence / OIS)

Upstream Regulator	Activation z-score	p-value of overlap
decitabine	2.058	1.16E-13
TGFB1	1.839	1.64E-12
Brd4	0.788	2.55E-13
TP53	0.653	1.44E-14
ERBB2	0.611	2.01E-13
forskolin	0.488	4.7E-13
D-glucose	0.271	1.45E-12
ERK	-0.193	1E-15
KRAS	-0.805	1E-13
EGF	-0.835	2.57E-16
lipopolysaccharide	-0.97	1.01E-13
HRAS	-3.116	1.09E-17

Co-culture
(Secondary senescence / OIS)

Upstream Regulator	Activation z-score	p-value of overlap
PD98059	4.571	5.34E-29
U0126	3.069	5.91E-29
dexamethasone	2.646	2.65E-33
TGFB1	2.622	1.3E-48
MYCN	-2.054	1.07E-22
Cg	-2.253	2.03E-24
EGF	-2.322	4.52E-22
EGFR	-2.617	3.39E-26
KRAS	-2.989	3.37E-24
PDGF BB	-3.101	4.29E-36
TNF	-3.435	2.34E-29
HRAS	-4.235	8.86E-37

Genes with adjusted p-value<0.05

B

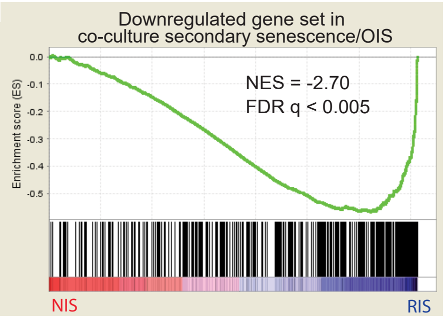

Leading edge genes: STC1, MMP1, SERPINB2, CDCP1, NRG1, SLC16A6, TFPI1, TMEM158, CSF3, IL8, MT2A, HMGA1, IL1B

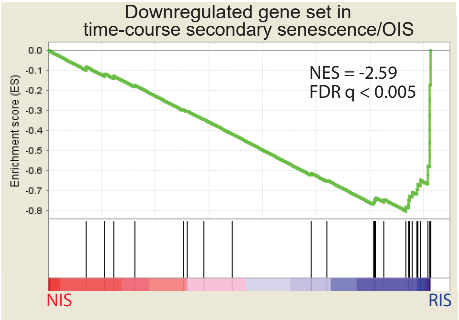

Leading edge genes: MMP3, STC1, MMP1, SERPINB2, SLC16A6, TFP12, IL2B,

C

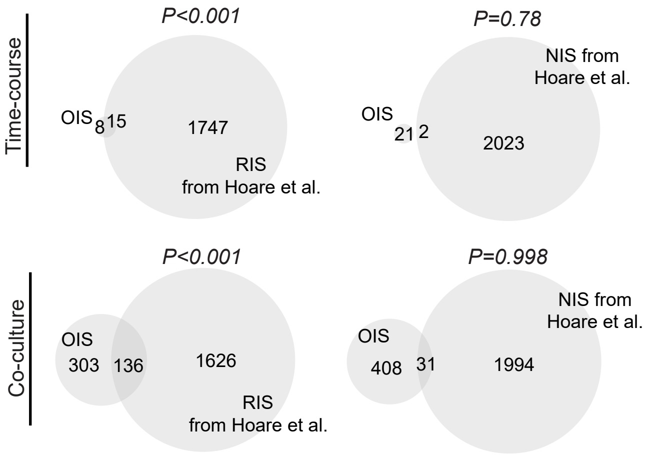

Figure S2, Related to Figure 2

(A) IPA analysis of the two senescence clusters from time-course and co-culture scRNA-seq. Red indicates activated upstream regulator and blue indicates inhibited upstream regulator.

(B) GSEA was used to assess the enrichment of secondary and primary senescence (OIS) DE genes in Hoare et al.'s NIS and RIS log2FC preranked genes. NES and FDR are shown.

(C) Venn diagrams overlapping expression signatures from top panel: time-course and bottom panel: co-culture experiments with NIS signature genes (OIS: OIS/Secondary senescence upregulated genes; NIS: Hoare et al.'s NIS/RIS upregulated genes; RIS: Hoare et al.'s RIS/NIS upregulated genes)

Figure S3

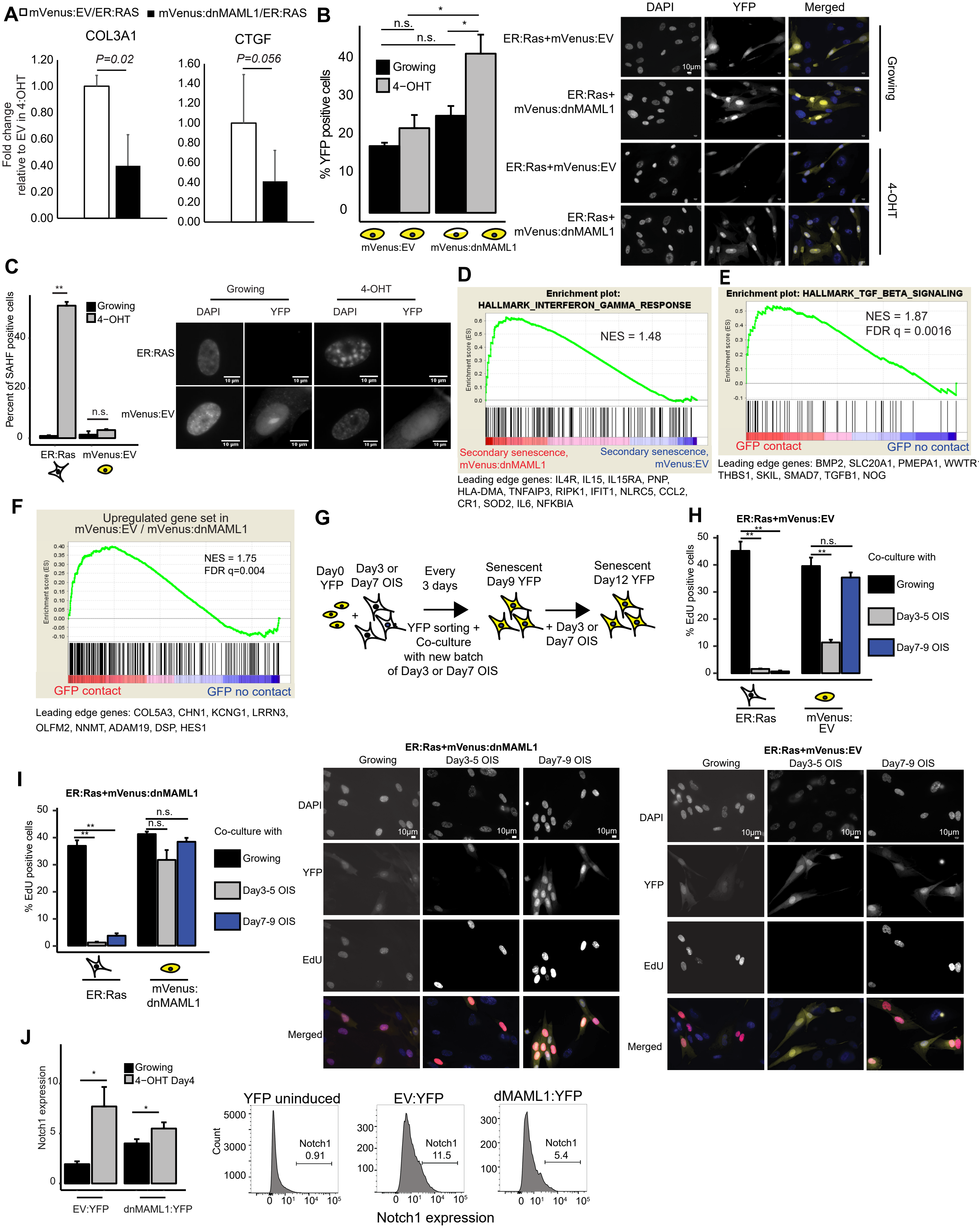

A
mVenus:EV/ER:RAS
mVenus:dnMAML1/ER:RAS
COL3A1
P=0.02
CTGF
P=0.056
Fold change relative to EV in 4:OHT
B
% YFP positive cells
Growing
4−OHT
mVenus:EV
mVenus:dnMAML1
DAPI
YFP
Merged
ER:Ras+mVenus:EV
ER:Ras+ mVenus:dnMAML1
C
Percent of SAHF positive cells
ER:RAS
mVenus:EV
D
Enrichment plot: HALLMARK_INTERFERON_GAMMA_RESPONSE
NES = 1.48
Secondary senescence, mVenus:dnMAML1
Secondary senescence, mVenus:EV
Leading edge genes: IL4R, IL15, IL15RA, PNP, HLA-DMA, TNFAIP3, RIPK1, IFIT1, NLRC5, CCL2, CR1, SOD2, IL6, NFKBIA
E
Enrichment plot: HALLMARK_TGF_BETA_SIGNALING
NES = 1.87
FDR q = 0.0016
GFP contact
GFP no contact
Leading edge genes: BMP2, SLC20A1, PMEPA1, WWTR1, THBS1, SKIL, SMAD7, TGFB1, NOG
F
Upregulated gene set in mVenus:EV / mVenus:dnMAML1
NES = 1.75
FDR q=0.004
GFP contact
GFP no contact
Leading edge genes: COL5A3, CHN1, KCNG1, LRRN3, OLFM2, NNMT, ADAM19, DSP, HES1
G
Day0 YFP
Day3 or Day7 OIS
Every 3 days
YFP sorting + Co-culture with new batch of Day3 or Day7 OIS
Senescent Day9 YFP
+ Day3 or Day7 OIS
Senescent Day12 YFP
H
ER:Ras+mVenus:EV
% EdU positive cells
Co-culture with
Growing
Day3-5 OIS
Day7-9 OIS
ER:Ras
mVenus: EV
I
ER:Ras+mVenus:dnMAML1
% EdU positive cells
Co-culture with
Growing
Day3-5 OIS
Day7-9 OIS
ER:Ras
mVenus: dnMAML1
DAPI
YFP
EdU
Merged
ER:Ras+mVenus:EV
J
Notch1 expression
Growing
4−OHT Day4
EV:YFP
dnMAML1:YFP
YFP uninduced
EV:YFP
dMAML1:YFP
Count
Notch1 0.91
Notch1 11.5
Notch1 5.4
Notch1 expression

Figure S3, Related to Figure 3

(A) Bar plot showing the expression of *CTGF* (n=6) and *COL3A1* (n=3) genes in EV or dnMAML1 cells compared to ER:Ras senescent cells by qPCR. (COL3A1: t=5.3405, df=2.4861, p=0.02; CTGF: t=2.2104, df=8.4894, p=0.056 using unpaired Student's t- test). Error bars represent SEM.

(B) Bar plot denoting the proportion of growing (black) or senescent (grey) mVenus cells with dnMAML1 or EV as proportion of all cells scored. Error bars are displayed as SEM; F[3,8] = 10.05, p<0.05 using one-way ANOVA with Tukey's test (n=3 for each condition). Representative images of mVenus cells and cells stained with DAPI are shown on the right. Scale bar 10μm.

(C) SAHF counts in OIS and secondary senescent (unpaired Student's t-test, ER:Ras t=-34.05, df=2.12, ** p<0.01; mVenus:EV t=-1.23, df=2.28, p=0.32; n=3 for each condition). Representative images are shown on the right.

(D) GSEA pre-ranked test for enrichment of interferon gamma response in mVenus:dnMAML1 identified as secondary senescence by scmap.

(E) GSEA pre-ranked test for enrichment of TGF-beta signalling in GFP contact compared to GFP no contact cells. mVenus:dnMAML1 identified as secondary senescence by scmap.

(F) GSEA pre-ranked test for enrichment of mVenus:EV signature genes in GFP contact/GFP no contact upregulated gene set.

(G) Schematic representation of co- culturing mVenus cells with Day 3 or Day 7 OIS cells.

(H) Bar plot showing EdU incorporation in OIS or mVenus:EV cells in growing (black), co- culture with Day 3 OIS (grey) or Day 7 OIS cells (blue). Error bars are displayed as SEM; F[5,18] = 144.4, p<0.001 using one-way ANOVA with Tukey's test (n=3 for each except for Day 3 OIS (n=6)). Representative images are shown below the bar plot. Scale bar 10μm.

(I) Bar plot showing EdU incorporation in OIS or mVenus:dnMAML1 cells in growing (black), co-culture with Day 3 OIS (grey) or Day 7 OIS cells (blue). Error bars are displayed as SEM. F[5,24] = 58, p<0.001 using one-way ANOVA with Tukey's test (n=3 for all conditions except for Day3 OIS (n=6)). **p<0.001, *p<0.05. Representative images are shown on the right of the bar plot. Scale bar 10μm. **j.** Barplot showing the upregulation of NOTCH1 on the cell surface of mVenus:EV and mVenus:dnMAML1 cells 4 days after co-culture with ER:Ras compared to non-co-cultured, growing mVenus:EV (mVenus:EV t = -3.27, df = 2.01, p-value = 0.041; mVenus:dnMAML1 t = -3.29, df =3.03, p-value = 0.023 using one-sided t- test). Error bars represent SEM. Representative FACs plots showing NOTCH1 staining of YFP uninduced fibroblasts and YFP:EV and YFP:dnMaml1 at 4 days of co-culture.

Figure S4

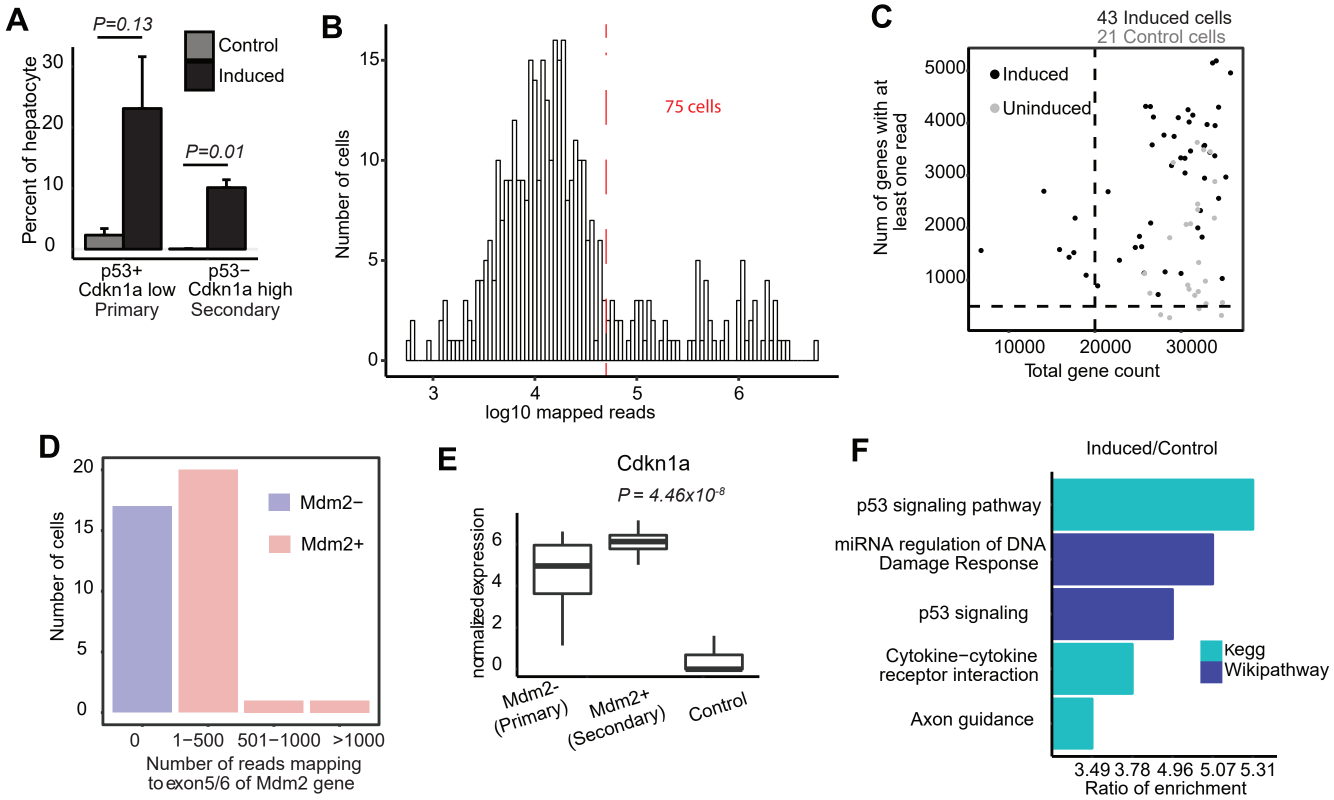

A
P=0.13
P=0.01
Control
Induced
Percent of hepatocyte
p53+
Cdkn1a low
Primary
p53−
Cdkn1a high
Secondary
B
Number of cells
75 cells
log10 mapped reads
C
43 Induced cells
21 Control cells
Induced
Uninduced
Num of genes with at least one read
Total gene count
D
Mdm2−
Mdm2+
Number of cells
0
1−500
501−1000
>1000
Number of reads mapping to exon5/6 of Mdm2 gene
E
Cdkn1a
P = 4.46x10-8
normalized expression
Mdm2- (Primary)
Mdm2+ (Secondary)
Control
F
Induced/Control
p53 signaling pathway
miRNA regulation of DNA Damage Response
p53 signaling
Cytokine−cytokine receptor interaction
Axon guidance
Kegg
Wikipathway
3.49 3.78 4.96 5.07 5.31
Ratio of enrichment

Figure S4, Related to Figure 4

(A) Bar graph denoting the percentage of primary and secondary hepatocytes (Primary: $t=2.4241$, $df=2.0641$, p-value = 0.1324; Secondary: $t=7.7563$, $df=2.0053$, $p=0.0161$ using unpaired Student's t-test).

(B) Histogram with the number of induced and control cells is plotted against log mapped reads. 75 single cells with at least 50,000 aligned reads are downsampled to 50,000 reads.

(C) Dot plot with the number of genes with at least one read to total gene count for induced (black) and control (grey) cells. Cells with a total gene count of more than 20,000 and 500 genes detected were retained.

(D) 17 *Mdm2*- cells were identified as cells with no reads mapping to exon5/6 of *Mdm2* gene and 22 *Mdm2*+ cells contained reads mapping to the exons.

(E) Box plots showing the expression of *Cdkn1a* in induced cells relative to control ($p=4.46\times10^{-18}$). The top and bottom bounds of the boxplot correspond to the 75 and 25th percentile, respectively. p-values were obtained using differential analysis in SCDE.

(F) Pathway analysis for induced/uninduced hepatocytes. Kegg pathways are shown in turquoise and Wikipathways in blue.
